# Aromatic residue Trp146 mediates dynamic interplay between type IVa pili and exopolysaccharide in social motility of *Myxococcus xanthus*

**DOI:** 10.1128/mbio.02568-25

**Published:** 2025-11-17

**Authors:** Yan Wang, Yipeng Wang, Weiwei Xue, Jiaxin Li, Fujian Zhang, Xiashi Lv, Fengyu Zhang, Yuezhong Li, Chuandong Wang, Wei Hu

**Affiliations:** 1 State Key Laboratory of Microbial Technology, Microbial Technology Institute, Shandong University, Qingdao, Shandong, China; 2 State Key Laboratory of Natural Medicines, Center of Drug Discovery, China Pharmaceutical University, Nanjing, China; South China Sea Institute of Oceanology, Guangzhou, Guangdong, China

**Keywords:** type IVa pili, exopolysaccharide, *Myxococcus xanthus*, social motility, glucosamine, tryptophan

## Abstract

The social motility (S-motility) of *Myxococcus xanthus* is driven by the intricate interplay between type IVa pili (T4aP) and exopolysaccharide (EPS). Although T4aP retraction furnishes the mechanical force necessary for cellular movement, EPS serves as a matrix for T4aP anchoring and instigates pilus retraction. Despite this interdependence, the molecular determinants governing specific T4aP-EPS recognition and interaction have yet to be fully elucidated. Utilizing a comprehensive approach, our study identifies glucosamine as the key monosaccharide within EPS that interacts with the major pilin PilA, mediated by a tryptophan residue (W146). Significantly, mutagenesis of W146 to alanine (W146A) resulted in a markedly impaired EPS recognition and abrogation of S-motility, although the T4aP retained the capacity for extension and retraction. Saturation mutagenesis studies further emphasized the critical role of aromatic conservation at PilA position 146 in ensuring effective EPS recognition. Given the pivotal roles of T4aP and EPS in orchestrating bacterial multicellular behavior, gaining a profound understanding of their interaction will provide fundamental insights into the molecular basis of the collective behavior within bacterial communities.

**IMPORTANCE:**

Our study reveals a key molecular mechanism that controls the interaction between T4aP and EPS, which is a critical process for bacterial social motility, biofilm formation, predation, and other collective behaviors. We identify a specific aromatic residue (W146) in the major pilin PilA that mediates direct binding to glucosamine-containing polysaccharides, thereby linking T4aP function and extracellular matrix recognition. This finding provides new insight into how bacteria use glycans to coordinate group behaviors within microbial communities. These results open potential strategies for controlling biofilm-related processes, such as disrupting infections or guiding beneficial microbial assemblies in environmental and industrial settings.

## INTRODUCTION

To ensure survival and reproductive success, microbial communities employ diverse regulatory mechanisms to coordinate collective cellular behaviors, which are essential for establishing and maintaining complex microbial ecosystems (1, 2). Among these systems, type IV pili (T4P)-related machinery, that is, type IVa pili (T4aP), type IVb pili (T4bP), type II secretion system (T2SS), Tad/Flp pili, and Com pili, plays critical roles in diverse functions such as motility, surface sensing, adherence, colonization, biofilm formation, genetic material uptake, and pathogenesis (3–6). Although T4P-like systems are ubiquitously distributed across nearly all major prokaryotic phyla and perform diverse functions, they universally depend on regulated cycles of pilus extension and retraction (7, 8). Particularly, interactions between T4P and extracellular components have emerged as key regulators of complex bacterial behavioral patterns (3). In the gram-negative soil bacterium *Myxococcus xanthus*, a T4aP-dependent social motility (S-motility) system coordinates group movement of cells on solid surfaces and underpins multicellular development and predation (9, 10), which is a mechanism mechanistically analogous to twitching motility in *Pseudomonas aeruginosa* and *Neisseria gonorrhoeae* (5). Functional studies demonstrate that S-motility is driven by T4aP extension/retraction cycles (11) and requires interactions between T4aP and exopolysaccharide (EPS) (12). Specifically, *M. xanthus* secretes EPS to guide the movement of neighboring cells (13), align cell bodies along the movement direction (14), and stimulate T4aP retraction (15), enabling rod-shaped cells to follow EPS trails deposited by predecessor cells. Remarkably, similar EPS trail-following behaviors have been observed during twitching motility in *P. aeruginosa* (16, 17).

The T4aP of *M. xanthus* assembles at the leading pole of each cell and anchors to either the substratum or the EPS secreted by neighboring cells (10). During retraction, individual T4aP filaments generate a pulling force of up to 150 pN to propel the cell forward (18). The ATPases PilB and PilT*,* members of the secretion ATPase superfamily, drive T4aP assembly and disassembly through ATP hydrolysis, respectively (19). The T4aP filament is primarily composed of the major pilin PilA (20, 21), which features a conserved N-terminal α-helix required for pilus core assembly and a C-terminal globular domain crucial for functional specificity (20, 22, 23). Additionally, a PilY1/minor pilin priming complex forms a kinked structure at the pilus tip (20, 24).

EPS, a cell-surface polysaccharide central to *M. xanthus* social behaviors (25, 26), is a heteropolymer containing unmodified, amino-derivatized, and N-acetylated monosaccharides (27–30). Beyond its role in S-motility, EPS contributes to extracellular matrix construction (31), intercellular adhesion (32), biofilm formation (33), and fruiting body development (34). EPS biosynthesis follows the Wzx/Wzy-dependent pathway, with core components encoded by the *eps* locus (29, 35, 36). Regulatory networks governing EPS production include the Dif chemosensory system (37–39), T4aP machinery (40, 41), second messenger c-di-GMP (42), and biosurfactant polysaccharide biosynthesis (29, 43).

Accumulating evidence indicates that *M. xanthus* self-produced EPS promotes S-motility through binding to PilA and stimulating T4aP retraction. Three lines of experimental evidence elucidate this EPS-T4aP functional interplay: PilA and T4aP specifically bind purified EPS *in vitro* (15, 44); exogenous EPS supplementation restores normal piliation levels in EPS-deficient mutants (e.g., *ΔdifA*) (15, 45); and intracellular pre-pilin accumulation sequesters EPS biosynthetic precursors, resulting in attenuated surface EPS production (40). In addition to surface motility, bacterial T4P directly engages with polysaccharides to mediate critical processes, including substrate binding, horizontal gene transfer, and host-pathogen adhesion (7, 46). Nevertheless, the molecular basis for polysaccharide specificity by T4P and its major pilins remains poorly understood. In this study, we combine bioinformatics and multilevel experimental approaches to demonstrate that a specific EPS monosaccharide and a conserved PilA residue are essential determinants of EPS-T4aP interactions in *M. xanthus*.

## RESULTS

### The C-terminal truncated variant PilACt of *M. xanthus* PilA exhibits intermediate binding affinity for EPS

**Fig 1:**
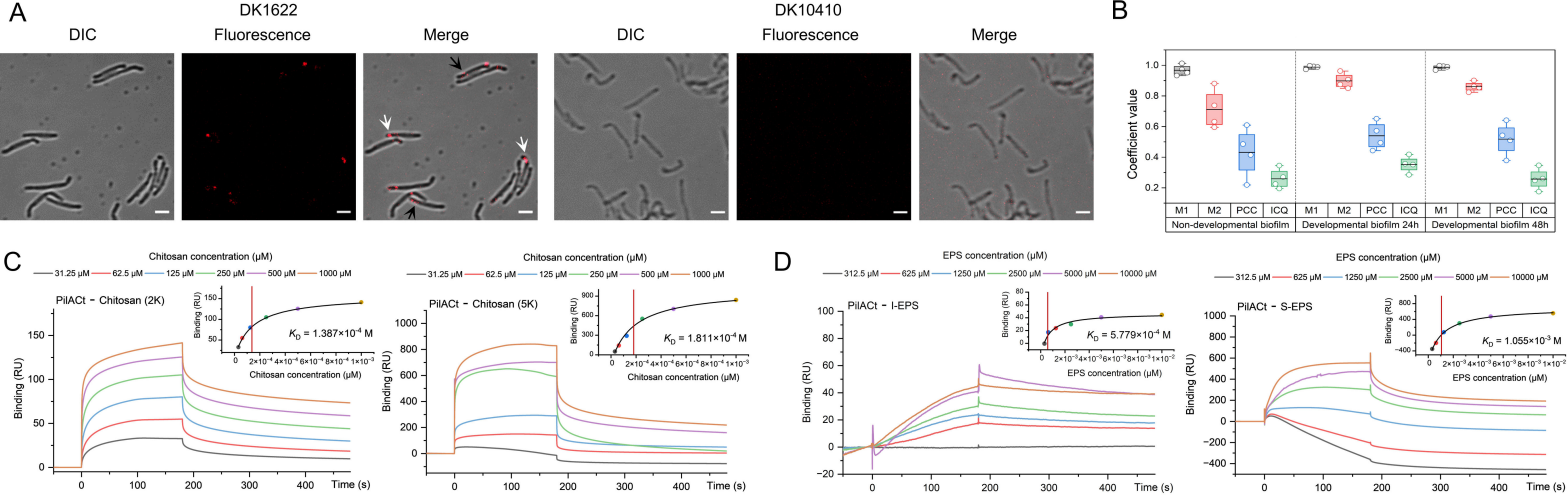
Moderate-affinity interaction between *M. xanthus* T4aP and EPS is mediated by PilA. (**A**) Immunolocalization of extracellular PilA in *M. xanthus* strains. Wild-type (DK1622) and Δ*pilA* (DK10410) cells were immunolabeled with rabbit anti-PilACt polyclonal antibodies, followed by Alexa Fluor 647-conjugated goat anti-rabbit IgG (red). Differential interference contrast (DIC) and fluorescence images were acquired under 63 × magnification. Scale bars represent 2 µm. The white and black arrows indicate the observed red fluorescent signals at the cell poles and non-polar regions, respectively. (**B**) Quantitative colocalization analysis of PilA (red) and EPS (blue) in DK1622 biofilms. Spatial correlation was evaluated using overlap coefficients M1 (PilA-EPS overlap) and M2 (EPS-PilA overlap), Pearson’s correlation coefficient (PCC), and intensity correlation quotient (ICQ), derived from fluorescence signals of PilA (red) and EPS (blue) in Fig. S1 (n = 4). (**C and D**) Surface plasmon resonance (SPR) binding kinetics. PilACt-chitosan (2 kDa and 5 kDa) interaction sensorgrams are shown in (**C**); results of PilACt binding to soluble EPS (S-EPS) and insoluble EPS (I-EPS) are shown in (**D**). Affinity constants (*K*_D_) were calculated using Biacore evaluation software (v3.0). Color-coded lines indicate analyte concentrations. RU: resonance units.

Previous studies have established that heterologous expression of full-length *M. xanthus* PilA is limited by its intrinsic insolubility (21, 44, 47). To circumvent this limitation, we generated a truncated variant (PilACt) spanning residues 32–208 (44), which retains the complete C-terminal globular domain essential for ligand recognition specificity (7). Building on the demonstrated capacity of eGFP-PilACt fusion proteins to label EPS (44), we developed rabbit anti-PilACt antibodies to investigate the spatial relationship between extracellular PilA and EPS in native *M. xanthus* DK1622 cells. In planktonic cultures, Alexa Fluor 647-based immunolabeling against PilACt exhibited specific polar fluorescence in a subset of WT cells, whereas the *ΔpilA* mutant (DK10410) completely lacked this signal (Fig. 1A). This partial labeling pattern is likely due to the detachment of T4aP (pilus shedding) during sample preparation. Accordingly, a portion of the red fluorescence observed in non-polar regions of the cells likely corresponds to shed pilus fragments attached to the cell surface. Red fluorescent signals localized at the cell poles exhibited a highly aggregated distribution, whereas those present in non-polar regions appeared as dispersed punctate patterns. Furthermore, the filamentous structure of the pili was not resolved in fluorescence images, which could be attributed to both limited resolution and pilus entanglement into clusters during the staining process. Biofilm architecture was analyzed using SYTO9 for cells (green) and Alexa Fluor 350-WGA for EPS (blue). Specific PilA signals were detected in both developmental and non-developmental biofilms (Fig. S1A), with control experiments confirming the absence of nonspecific secondary antibody binding (Fig. S1B). In merged images, the overlap of red (PilA/T4aP) and blue (EPS) channels produced purple zones, indicating colocalization—a finding further supported by quantitative colocalization analysis (Fig. 1B). Across all biofilm structures, high M1 values indicated that a substantial portion of PilA signal overlapped with EPS, while lower M2 values suggested that EPS was more broadly distributed than PilA. Both Pearson’s correlation coefficient (PCC) and the intersection coefficient quotient (ICQ) returned positive values, confirming a statistically significant correlation between PilA and EPS signals. High-magnification views further revealed intimate associations between EPS and both cell-attached and shed pili. These results align with prior studies  (21, 44) and collectively support the occurrence of direct T4aP-EPS interactions under native conditions.

*In vitro* co-precipitation assays followed by immunoblotting with anti-PilACt antibodies demonstrated that PilACt retained EPS-binding ability comparable with full-length PilA (Fig. S2). We therefore employed surface plasmon resonance (SPR) on a Biacore system to quantitatively evaluate EPS-PilA binding (48), using immobilized PilACt as the ligand. Sensorgrams generated from soluble chitosan (2 kDa and 5 kDa, based on its established interaction with T4aP (15, 45) at stepwise gradient concentrations revealed characteristic association/dissociation curves, with resonance unit (RU) increases confirming successful PilACt immobilization and ligand binding (Fig. 1C). Quantitative analysis showed 2 kDa chitosan bound PilACt with an apparent affinity (*K*_D_) of 1.387 × 10^−4^ M, and 5 kDa chitosan exhibited a *K*_D_ of 1.811 × 10^−4^ M. Soluble EPS (S-EPS) extracted from the DK1622 extracellular matrix displayed concentration-dependent interactions with PilACt (*K*_D_ = 1.055 × 10^−3^ M, Fig. 1D). Notably, insoluble EPS (I-EPS) exhibited similar binding affinity (*K*_D_ = 5.779 × 10^−4^ M). The observed micromolar-range affinities suggest dynamic, physiologically relevant T4aP-EPS interactions, consistent with reversible binding requirements for coordinated motility in *M. xanthus* (49). Due to the insoluble nature of I-EPS and the risk of clogging the microfluidic channels, it was excluded from further SPR analyses.

### Glucosamine mediates specific interactions between EPS and PilA in *M. xanthus*

*M. xanthus* EPS comprises a heteropolymer of unmodified monosaccharides (arabinose, galactose, glucose, mannose, rhamnose, xylose), amino sugars (glucosamine, GlcN), and N-acetylated derivatives (N-acetyl-glucosamine, GlcNAc; N-acetyl-mannosamine, ManNAc) (27–30). Western blot analysis of surface pili demonstrated that GlcN and GlcNAc induce hyperpiliation phenotypes in *M. xanthus* DK1622 cells, suggesting that these two monosaccharides act as key molecular cues for T4aP-mediated retraction (15). To investigate the specificity of sugar-PilA interactions, we conducted systematic binding assays between EPS monosaccharides and the C-terminal domain of PilA (PilACt) using SPR and isothermal titration calorimetry (ITC).

**Fig 2:**
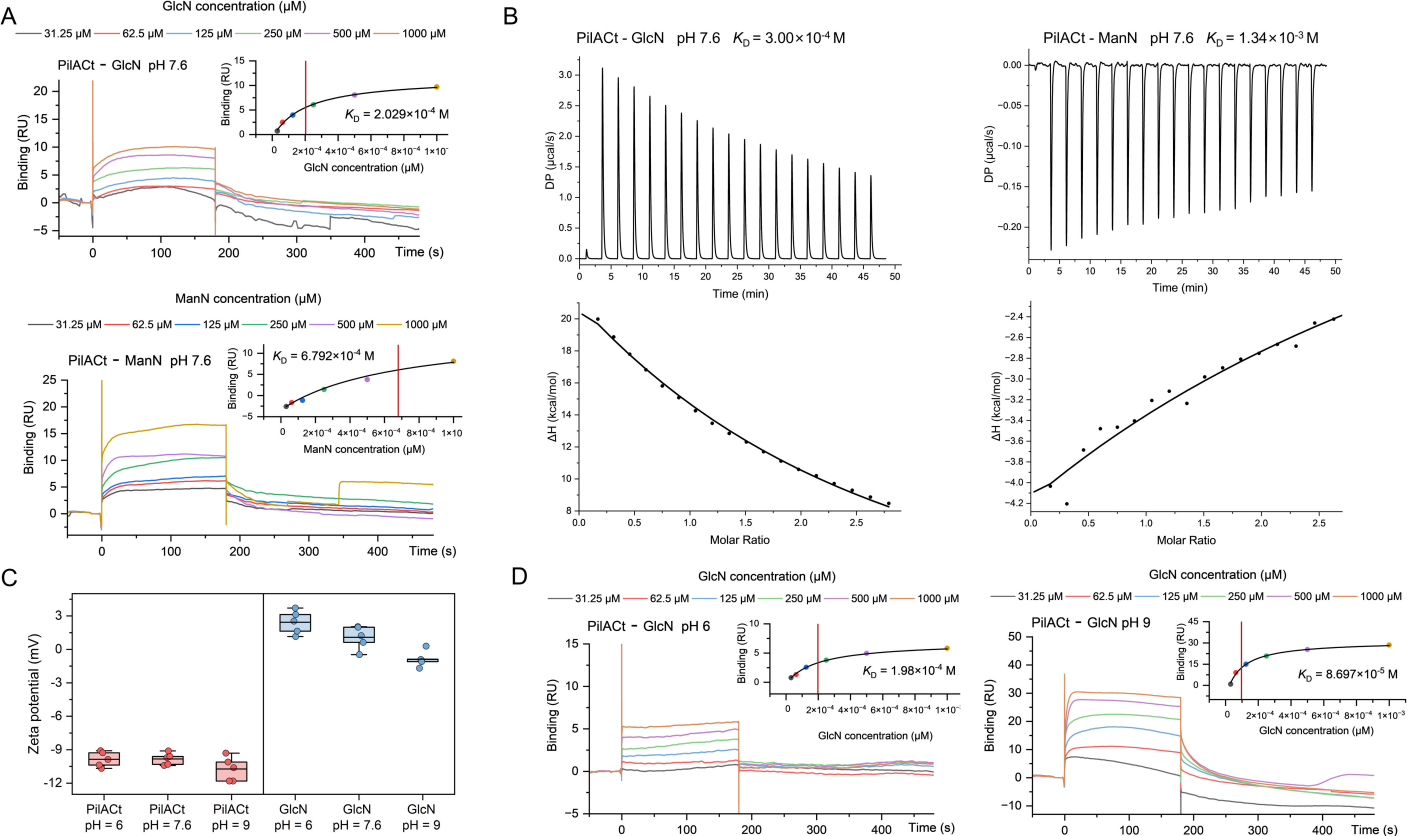
PilACt preferentially binds glucosamine (GlcN) over mannosamine (ManN) via non-electrostatic mechanisms. (**A**) SPR sensorgrams of PilACt-GlcN/ManN interactions at physiological pH 7.6. (**B**) ITC thermodynamic profiling. Top: Raw heat flux during sequential GlcN/ManN injections into PilACt. Bottom: Normalized enthalpy changes (*ΔH*) with one set of sites model fits (black lines). (**C**) Surface charge dynamics. Zeta potentials of PilACt (left) and GlcN (right) across pH 6-9 (*n* = 5). (**D**) pH-dependent binding kinetics. SPR sensorgrams comparing GlcN-PilACt interactions at acidic (pH 6) and alkaline (pH 9) conditions. *K*_D_ is annotated in the upper right. Color-coded lines indicate analyte concentrations. RU: resonance units.

SPR sensorgrams revealed no measurable binding of unmodified monosaccharides (Fig. S3A) or N-acetylated derivatives (Fig. S4A) to PilACt, as indicated by response units remaining near baseline across tested concentrations (31.25–1,000 µM). ITC power compensation analyses (50) confirmed negligible enthalpy changes during titrations, ruling out nonspecific binding events (Fig. S3B and S4B). In stark contrast, GlcN exhibited concentration-dependent binding to PilACt, with SPR response units increasing proportionally to injected concentrations (Fig. 2A, top panel). ITC thermogram (Fig. 2B, left panel) demonstrated exothermic binding with a 1:1 stoichiometry (*n* = 1.47) and moderate binding affinity (*K*_D_ = 3.00 × 10^−4^ M). Given the presence of ManNAc in *M. xanthus* EPS, we further analyzed its deacetylated derivative, mannosamine (ManN). ManN displayed weaker binding (Fig. 2A bottom panel and 2B right panel, *K*_D_ = 1.34 × 10^−3^ M), highlighting PilACt’s preferential recognition of GlcN.

Furthermore, electrostatic profiling under varying pH conditions (pH 6–9) revealed unexpected binding behavior. As shown in Fig. 2C, zeta potential measurements showed that GlcN’s surface charge decreased from +2.43 mV (pH 6) to −0.87 mV (pH 9), whereas PilACt maintained negative surface charge (-9.9 to −10.7 mV). Despite both molecules being negatively charged at pH 9.0, the strongest binding affinity (*K*_D_ = 8.697 × 10^−5^ M) was observed at this pH, with moderate affinity retained at physiological pH 7.6 (Fig. 2D). These results collectively indicate that GlcN recognition by PilA is predominantly governed by non-electrostatic interactions, likely involving hydrogen bonding and hydrophobic forces, which facilitate EPS-T4aP complex formation during social motility.

### W146 is a critical residue mediating PilA-EPS interactions

**Fig 3:**
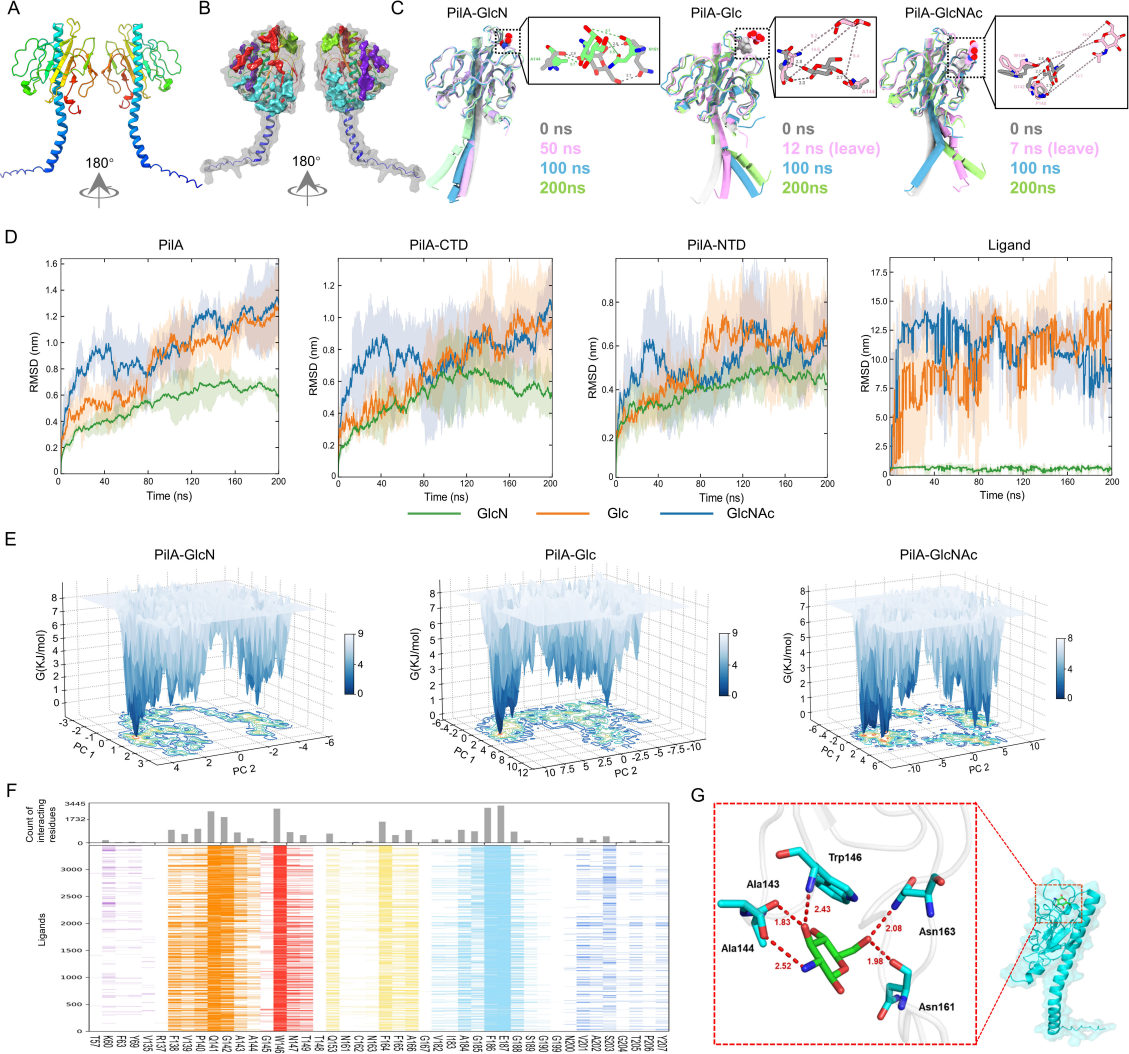
Integrated computational analysis identifies key residues in *M. xanthus* PilA mediating GlcN recognition. (**A**) Predicted 3D structure of PilA. Cartoon representation of AlphaFold2-derived structure: N-terminal (including the signal peptide) highlighted in blue and the C-terminal in red. (**B**) Ligand-binding pocket prediction. SiteMap analysis identified four putative binding sites: Space 1 (green), Space 2 (red), Space 3 (cyan), and Space 4 (purple). PilA surface model (gray, 70% transparency) illustrates pocket accessibility. (**C**) Ligand binding stability analysis. Overlay of representative snapshots from a 200-ns molecular dynamics (MD) simulation of PilA with GlcN, glucose, and GlcNAc, respectively. In the PilA-GlcN complex, GlcN maintained stable positioning within the binding pocket throughout the simulation period, as shown by consistent ligand localization (sphere representation). Conversely, in PilA-glucose and PilA-GlcNAc systems, both ligands (gray spheres) initially occupied positions corresponding to the putative binding pocket but exhibited progressive displacement, ultimately dissociating completely from the binding pocket (colored spheres). Inset: Close-up view of PilA with the ligand interactions. Gray represents the 0 ns position, and color indicates either 200 ns or when the ligands dissociate from the binding pocket. The hydrogen bonds are represented by dotted lines. Distances shown are in Å. (**D**) Conformational stability analysis. Root-mean-square deviation (RMSD) trajectories from 200-ns MD simulations assess the structural fluctuations of PilA, GlcN, glucose, and GlcNAc. Shaded areas represent the standard error of the mean (SEM) calculated from three independent replicates. CTD: C-terminal domain; NTD: N-terminal domain. (**E**) Free energy landscape (FEL) analysis. Principal component analysis (PCA)-derived FELs characterize the thermodynamic stability of PilA in complex with GlcN, glucose, and GlcNAc, respectively. Conformational dynamics were quantified using the first two principal components (PC1 and PC2), projected as three-dimensional contour plots. The Gibbs free energy gradient (kJ/mol) is represented by a color scale, with white regions denoting high-energy states. (**F**) Interaction histogram. Induced fit docking (IFD) of 3445 PilA-GlcN conformations. Colored lines denote each contact of a particular residue with GlcN; the histogram shows interaction frequency. (**G**) High-affinity binding mode. Optimized docking pose highlights GlcN (stick) hydrogen-bonded to key residues (A143/A144/W146/N161/N163). Inset: Zoomed view of binding pocket (red dashed box). Distances shown are in Å.

To decipher the molecular basis of T4aP-EPS binding in *M. xanthus*, we sought to identify key PilA residues governing EPS recognition. Given the lack of an experimentally resolved PilA structure for *M. xanthus* at the study’s initiation, we employed AlphaFold2 (51) to predict a three-dimensional (3D) PilA model. As shown in Fig. 3A, the predicted structure displayed a characteristic architecture featuring an elongated N-terminal α-helix connected to a globular C-terminal domain, which harbored a central β-sheet core flanked by surface-exposed loops. Structural validation via Ramachandran plot analysis (Fig. S5) showed that 85.9% residues were in favored regions (14.1% in allowed regions; 0% in disallowed regions). Following the recent release of a 3.0 Å cryo-EM PilA structure (23), we structurally aligned our model with PDB 8TJ2 using US-align (Fig. S6). The TM-score (ranging from 0 to 1) quantified structural similarity, revealing high accuracy (TM-score = 0.912) for the predicted conformation. Discrepancies localized to five unstructured residues near P22 in the α1-helix, likely attributable to modeling monomeric PilA versus assembled T4aP filaments. Notably, the C-terminal domain (i.e., PilACt) exhibited exceptional congruence (TM-score = 0.950), validating its structural reliability for subsequent analyses.

Four putative ligand-binding pockets were identified in the PilA model (Fig. 3B). Blind docking of 2 kDa chitosan (an EPS analog) to these pockets was performed using AutoDock Vina (52). Ten top-ranked poses were selected based on binding affinity and root mean square deviation (RMSD) clustering (Fig. S7; Table S2). The lowest-energy conformation (Model I) formed hydrogen bonds with residues D71, A92, P140, W146, L150, T155, I156, and C159 (Fig. S7).

Molecular dynamics (MD) simulation was employed to assess the stability of Model I in the PilA-chitosan complex. The complex conformation stabilized after 10 ns, with subsequent RMSD fluctuations averaging ~0.5 nm (Fig. S8A). Elevated RMSD values in both PilA and the PilA-chitosan complex primarily originated from enhanced flexibility in loop regions, as further corroborated by root-mean-square fluctuation (RMSF) analysis (Fig. S8B), which showed increased mobility for residues within these loops. To quantify residue-specific contributions to the binding, we performed MM-PBSA-based binding free energy calculations and per-residue free energy decomposition (Fig. S8C). Key energetic contributors (A92, P140, W146, T148, T149, V152, and T155) were predominantly localized within a single predicted binding pocket (green-labeled region in Fig. 3B), which was designated as the dominant binding pocket for further investigation.

A 200-ns MD simulation focused on the dominant PilA binding pocket (green region in Fig. 3B) revealed distinct ligand-binding behaviors. Conformational snapshots analyzed at four time points (0, 50, 100, and 200 ns, Fig. 3C) demonstrated that the PilA-GlcN complex maintained exceptional structural stability, with RMSD values consistently ranging between 0.1 and 0.5 nm (Fig. 3D; Movie S1), indicating minimal conformational rearrangements. In contrast, glucose dissociated rapidly from the binding pocket within 12 ns, accompanied by pronounced RMSD fluctuations. Comparative analysis showed significantly greater structural deviation in PilA-glucose complexes (PilA RMSD ~1.4 nm relative to the initial conformation; Fig. 3C and D; Movie S2). Similarly, the PilA-GlcNAc complex exhibited progressive expansion of the binding pocket, leading to unstable ligand positioning characteristic of transient interactions (Fig. 3D; Movie S3). Notably, RMSD fluctuations in PilA were primarily localized to the C-terminal domain.

Principal component analysis (PCA) of PilA-GlcN/Glc/GlcNAc complexes (Fig. 3E) characterized conformational dynamics through free energy landscape (FEL) profiling. Gibbs free energy values (0–9 kJ/mol) identified distinct conformational states, with the lowest-energy basins corresponding to thermodynamically stable configurations, most prominent in the PilA-GlcN system. PilA-Glc and PilA-GlcNAc complexes exhibited broader energy basins, with ΔG values indicating distributed local minima across wider free energy ranges. Although PilA-GlcN minima were separated by substantial energy barriers, those in PilA-Glc/GlcNAc systems were closely spaced, suggesting lower energy requirements for conformational transitions. These dynamic observations directly corroborated SPR and ITC data, establishing GlcN as the critical EPS monosaccharide mediating stable PilA interactions.

Induced fit docking (IFD) of 3445 GlcN-PilA conformations identified six key interaction residues (Q141, G142, W146, F164, F186, and E187) via cluster analysis (Fig. 3F). The highest-affinity pose revealed a hydrogen-bond network between GlcN and A143, A144, W146, N161, and N163 (mean bond length = 2.17 Å; Fig. 3G). Consistent with the established glycan recognition mechanisms (53, 54), aromatic residues (Y69, W146, F164, W181, and F186) in PilA were prioritized for functional validation. Although AlphaFold-Multimer failed to generate a complete and reliable T4aP structure comprising 4-10 PilA monomers that satisfied previously established structural parameters (20), it was observed that the relative positions of three side chains were highly consistent in both the seven-subunit (PilA-7, Chain 7-A-E-D) and ten-subunit (PilA-10, Chain A-B-J) pili complexes, suggesting analogous organization in native T4aP filaments (Fig. S9). This conserved local structural motif served as a key internal reference for subsequent global assembly using Rosetta docking. Subsequently, Rosetta’s symmetric docking with sparse constraints (55, 56) generated assembled *M. xanthus* DK1622 T4aP filament models, with predicted binding sites mapped to solvent-exposed regions (Fig. S10). Alignment of this predicted T4aP model with the cryo-EM structure (23) yielded high accuracy (TM-score = 0.917; Fig. S6B). Strikingly, W146 occupied a solvent-accessible position on the pilus periphery (Fig. S10B). Given the high conservation of tryptophan in bacterial chitin-binding domains (57), W146 was hypothesized to serve as the primary EPS recognition site in *M. xanthus* PilA.

**Fig 4:**
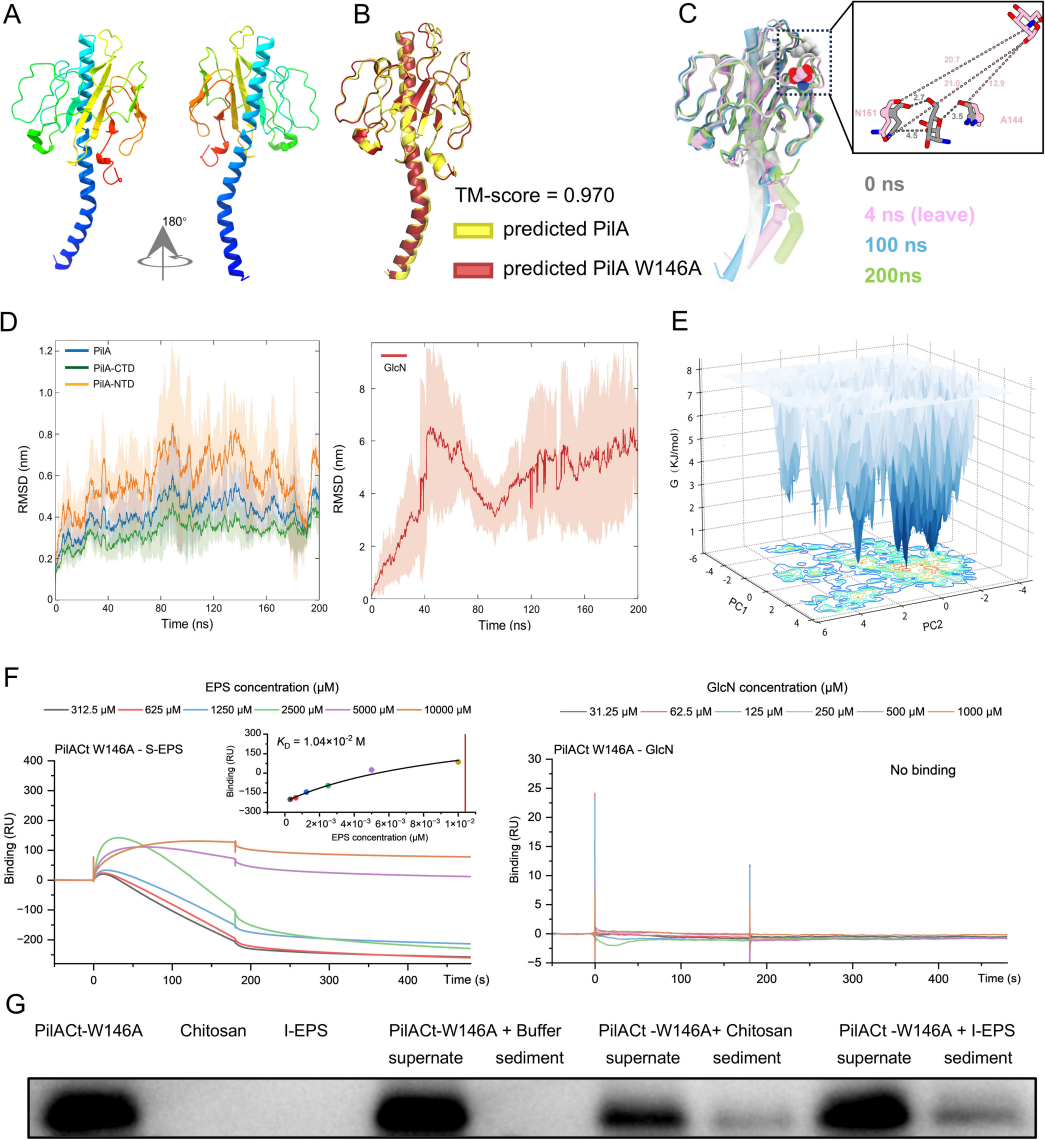
W146A substitution diminishes PilA-EPS/GlcN *in vitro* interactions. (**A**) Predicted 3D structure of PilA-W146A. The N-terminal was highlighted in blue and the C-terminal in red. (**B**) Structural congruence analysis between predicted PilA (yellow) and predicted PilA-W146A (red). (**C–D**) Molecular dynamics analysis of the PilA-W146A-GlcN complex. (**C**) GlcN binding stability with PilA-W146A. Overlay of representative snapshots from a 200-ns simulation of PilA-W146A with GlcN. The initial position of GlcN is shown as gray spheres, and the position at 200 ns is shown as colored spheres. Inset: Close-up view of PilA-W146 with GlcN interactions. The hydrogen bonds are represented by dotted lines. Distances shown are in Å. (**D**) Conformational stability analysis of PilA-W146A-GlcN complex. Shaded areas represent SEM derived from three independent replicates. CTD: C-terminal domain; NTD: N-terminal domain. (**E**) FEL analysis of PilA-W146A-GlcN complex. Conformational dynamics were quantified using the first two principal components (PC1 and PC2), projected as three-dimensional contour plots. The Gibbs free energy gradient (kJ/mol) is represented by a color scale, with white regions denoting high-energy states. (**F**) SPR binding kinetics. Sensorgram of PilACt-W146A interacting with S-EPS or GlcN. Representative sensorgram and calculated *K*_D_ values are shown. Color-coded lines indicate analyte concentrations. RU: Resonance units. (**G**) Precipitation of PilACt-W146A by *M. xanthu*s I-EPS or chitosan *in vitro*. Anti-PilACt antibodies (1:5,000 dilution) were used for immunodetection by western blot.

To functionally validate the role of W146, a W146A substitution mutant (PilA-W146A) was generated through AlphaFold2-based structural modeling of PilA (Fig. 4A). Structural alignment confirmed the mutant retained a tertiary structure indistinguishable from wild-type PilA (WT PilA; Fig. 4B), indicating the W146A substitution caused no global structural perturbations. Consistent RMSD trajectories (below 0.5 nm) over the 200-ns simulation further demonstrated structural integrity maintenance. However, the mutation induced localized instability, manifested by premature GlcN dissociation (4 ns) and excessive ligand RMSD fluctuations (>6 nm; Fig. 4C and D; Movie S4). FEL analysis revealed closely distributed local minima for PilA-W146A (Fig. 4E), indicating reduced energy barriers for conformational transitions compared with WT. To quantify binding effects, a single-residue PilACt variant (PilACt-W146A) was analyzed for EPS/GlcN affinity. SPR assays showed a 10-fold increase in *K*_D_ value (1.040 × 10^−2^ M of PilACt-W146A versus 1.055 × 10^−3^ M of wild-type PilACt) for S-EPS binding, whereas GlcN binding became undetectable (Fig. 4F). Co-precipitation experiments corroborated these findings, showing markedly reduced interactions of PilA-W146A with both I-EPS and insoluble chitosan relative to WT (Fig. 4G and 1C). To assess broader aromatic residue contributions, four additional PilA aromatic residues (Y69, W181, F164, and F186) were individually substituted with alanine. The PilACt-F164A mutant underwent intracellular degradation, likely due to structural instability, precluding heterologous expression. In contrast, PilACt-Y69A, PilACt-W181A, and PilACt-F186A exhibited S-EPS/GlcN binding affinities indistinguishable from WT (Fig. S11). Collectively, these data establish tryptophan residue W146 as critical for mediating PilA-EPS interactions.

### *M. xanthus* PilA-W146A mutation exhibits retractable T4aP and impaired EPS recognition

**Fig 5:**
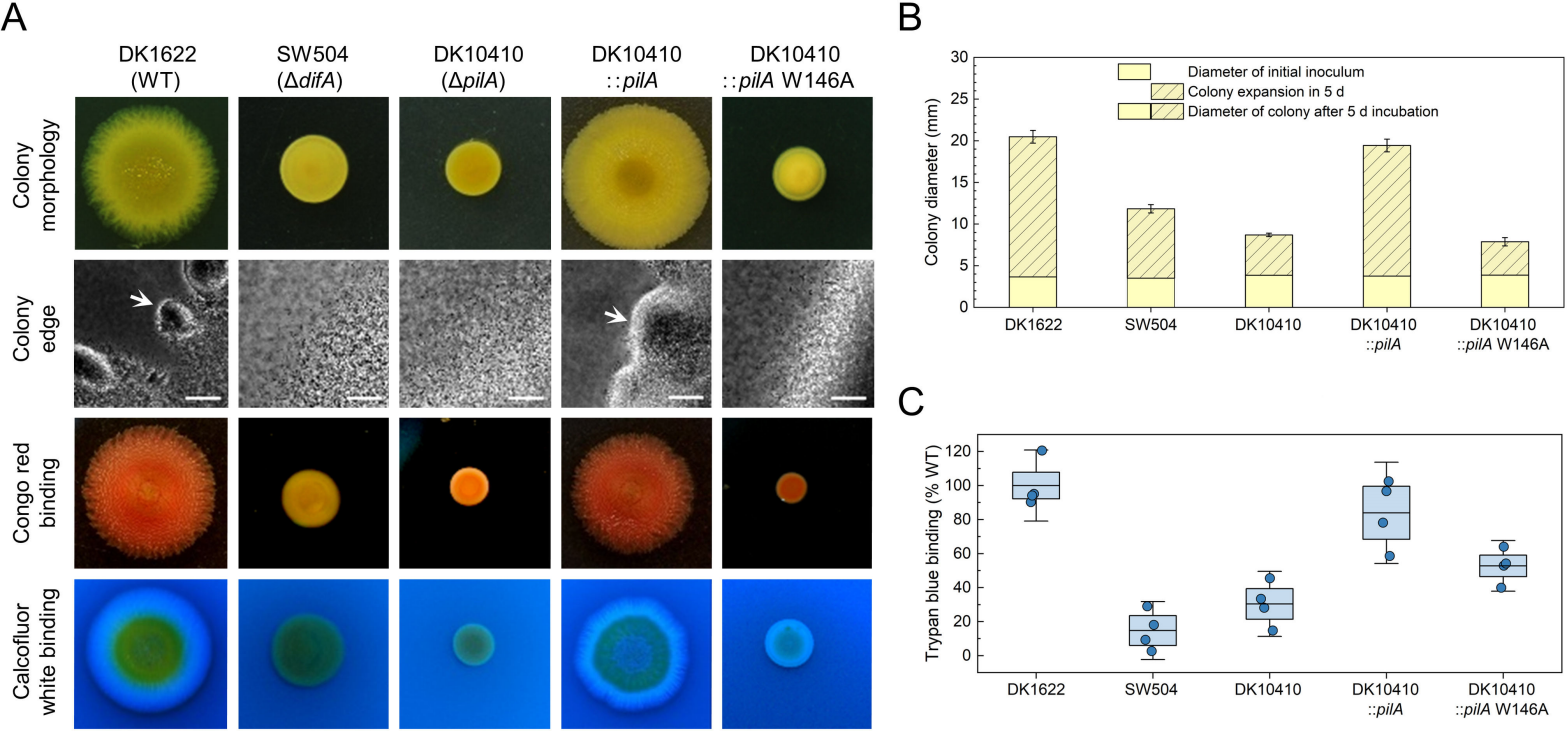
PilA-W146A mutant exhibited defects in S-motility-mediated colony expansion and reduced EPS production. (**A**) Representative images showing colony expansion, Congo red binding, and calcofluor white binding after 5-day incubation on 0.3% CTT agar. The edge of colony expansion was observed under a microscope using a 20 × objective lens, and scale bars represent 50 µm. Strains motile by T4aP generate flares at the colony edge, indicated by white arrows. (**B**) Colony expansion diameters. Expanded colonies (yellow bars) are defined as an increase in colony diameter (*n* = 3). (**C**) Spectrophotometric quantification of trypan blue binding ability by EPS, expressed as percentage of WT EPS-dye complexes after background subtraction (*n* = 4).

**Fig 6:**
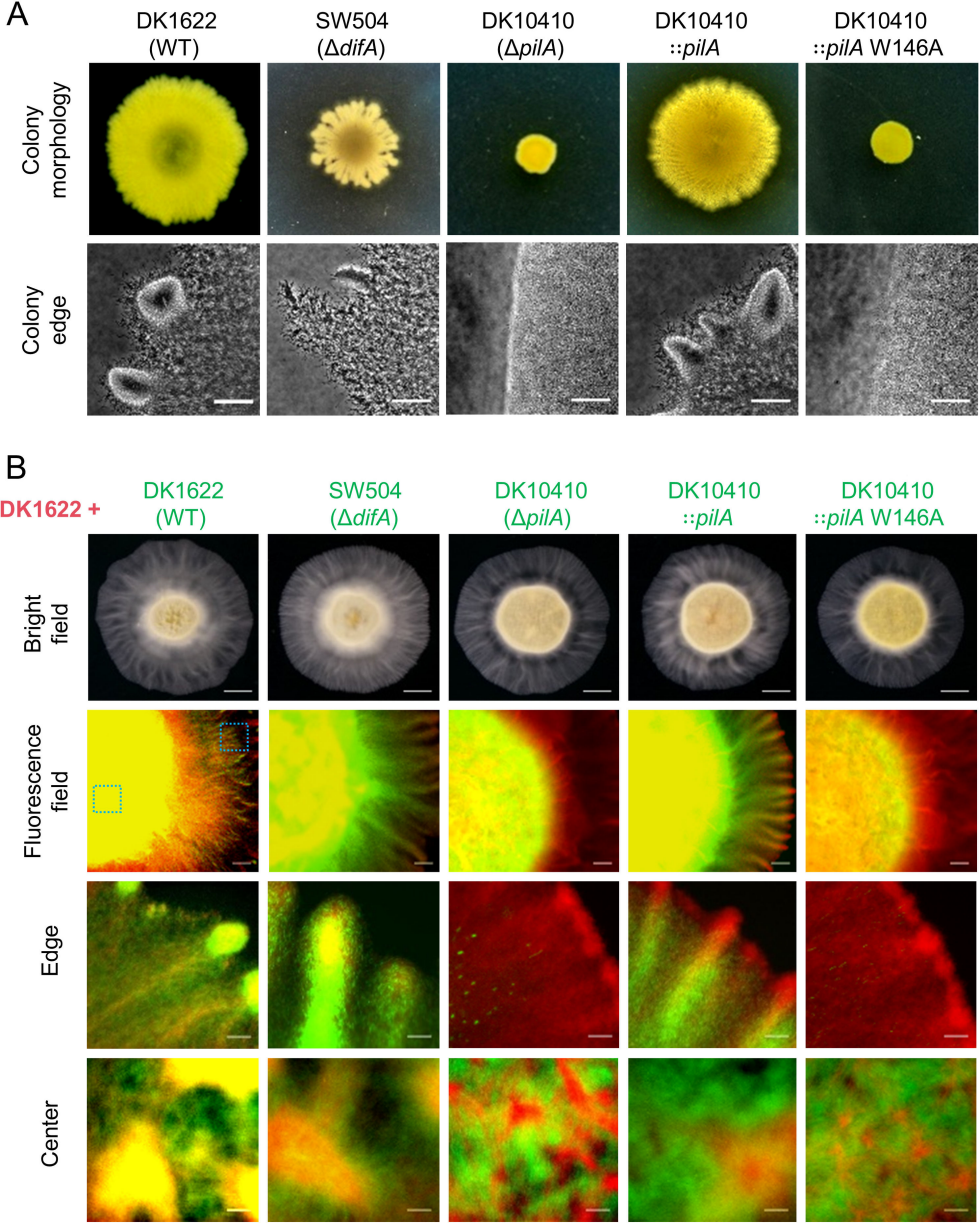
Exogenous EPS fails to rescue S-motility defects in PilA-W146A. (**A**) EPS supplementation assay. Top: Representative images of swarm morphologies on 0.3% CTT agar supplemented with purified EPS after 5-day incubation (*n* = 3). Bottom: Phase-contrast micrographs (20×) of colony edges (scale bars = 50 µm). (**B**) Mixed population analysis. First panel: Stereomicroscopy of mixed colonies (1:1 WT-mCherry/mutant-eGFP) after 24 h (scale bar = 1 mm). Second panel: Fluorescence overlay (4 × objective) showing spatial segregation (scale bar = 100 µm). Last two panels: magnified views (20 × objective) of the colony edge and center, as indicated by the green boxes in the second panel (scale bar = 20 µm).

To elucidate the *in vivo* functional significance of W146 in PilA, a W146A mutant strain (DK10410::*pilA* W146A, hereafter W146A mutant) was generated by complementing the pilA-deficient strain DK10410 (∆*pilA*) with the full-length pilA gene encoding a single-residue substitution (W146A). Comparative analysis of colony expansion dynamics revealed that after 5-day incubation on 0.3% agar, the W146A mutant exhibited severely attenuated S-motility, with expansion capacity reduced to ~20% of WT levels and phenocopying the *ΔpilA* control (Fig. 5A and B). Coordinated group migration from colony edges (as indicated by white arrows in Fig. 5A), a hallmark of functional S-motility, was absent in both *ΔpilA* and W146A mutants, mirroring the EPS-deficient strain SW504 (*ΔdifA*). According to the previous reports establishing EPS as an essential extracellular matrix component for S-motility (15, 39), quantitative assessment through trypan blue binding assays (Fig. 5C) showed that the W146A mutant produced approximately 50% of WT EPS levels, slightly higher than *ΔdifA* (~20%) and *ΔpilA* (~30%) of severely EPS-defects. Chromogenic differentiation on Congo red and calcofluor white (CFW) agar further confirmed this partial EPS deficiency, with W146A colonies exhibiting stronger dye binding than *ΔdifA* and *ΔpilA* but weaker than WT (Fig. 5A). To decouple EPS deficiency and T4aP dysfunction, we performed two critical experiments, that is, EPS supplementation assay and mixed population analysis. First, the purified EPS was pre-supplemented onto the surface of soft agar prior to inoculation, and exogenous EPS partially restored *ΔdifA* S-motility but failed to rescue *ΔpilA* or W146A mutant (Fig. 6A). Second, spatial tracking of eGFP-labeled mutants co-inoculated with mCherry-WT cells (1:1 ratio) revealed distinct niche partitioning (Fig. 6B). Although *ΔdifA* cells achieved peripheral localization through WT-produced EPS hijacking (edge occupancy ~48%), both W146A (edge occupancy ~0.03%) and *ΔpilA* (edge occupancy ~0.01%) remained restricted to the colony core, demonstrating inability to exploit shared EPS resources. These results indicate that the S-motility defect in W146A extends beyond a reduction in EPS production.

**Fig 7:**
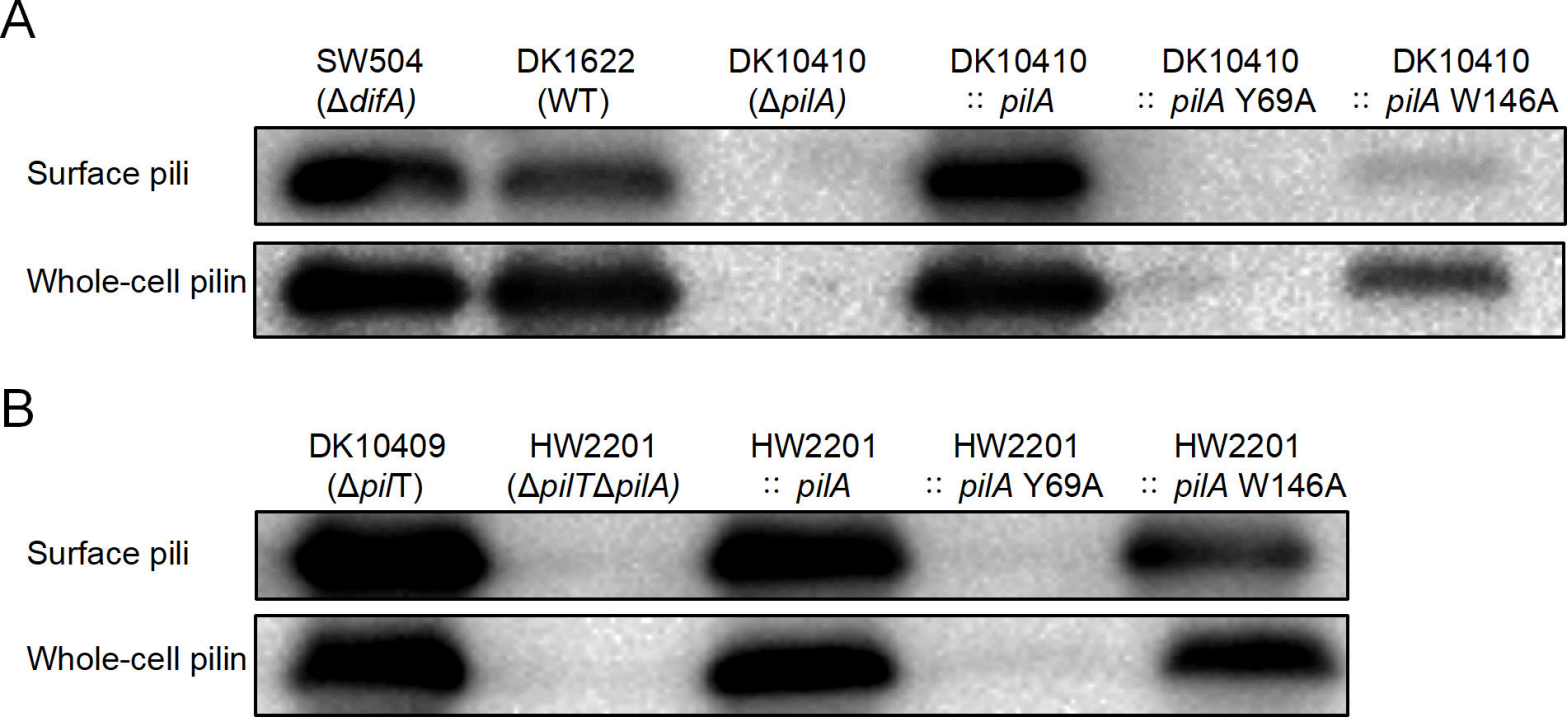
Mutational analysis reveals distinct pilus assembly mechanisms in PilA-Y69A and PilA-W146A variants. The detection of surface pili and whole-cell pilin for PilA variants Y69A and W146A was conducted using western blot analysis probed by anti-PilACt antibodies (1:5,000 dilution) in the genetic background of (**A**) *pilA* deletion and (**B**) *pilApilT* double deletion.

Contrary to the initial hypothesis that the W146A mutation would abolish PilA production in DK1622, as previously observed for many pilin mutations (40), immunoblot analysis revealed that the W146A mutant retained the ability to produce surface-associated pili and whole-cell pilin, albeit at reduced levels (~14% of WT, Fig. 7A). This partial piliation phenotype sharply contrasted with the null control strain DK10410::*pilA* Y69A, in which neither surface pili nor whole-cell pilin were detectable (Fig. 7A, lane 5). The residual EPS production observed in the W146A mutant (Fig. 5C) is likely attributable to this low level of surface piliation, given the established role of surface pili as regulators of EPS synthesis (22, 41).

The W146A mutant exhibited significantly fewer surface pili compared with wild type, a defect that could stem from impairments in T4aP assembly, extension, or retraction. To further investigate this, PilA variants were expressed in a *ΔpilTΔpilA* double-mutant background (strain HW2201). Since PilT is an ATPase responsible for T4aP retraction, and previous studies have shown that hyper-retraction phenotypes can be suppressed by pilT deletion (41, 58, 59), we assess piliation in this context. Consistent with these reports, deletion of *pilT* in the W146A background (HW2201::*pilA* W146A) restored surface piliation to approximately 57% of the *ΔpilT* control level (Fig. 7B), indicating W146A pilin subunits retain polymerization competence and that the hyper-retraction phenotype is PilT-dependent. Therefore, we propose that the reduced surface piliation in the W146A mutant results from enhanced PilT-mediated retraction, leading to fewer pili being exposed on the cell surface at the time of sampling.

**Fig 8:**
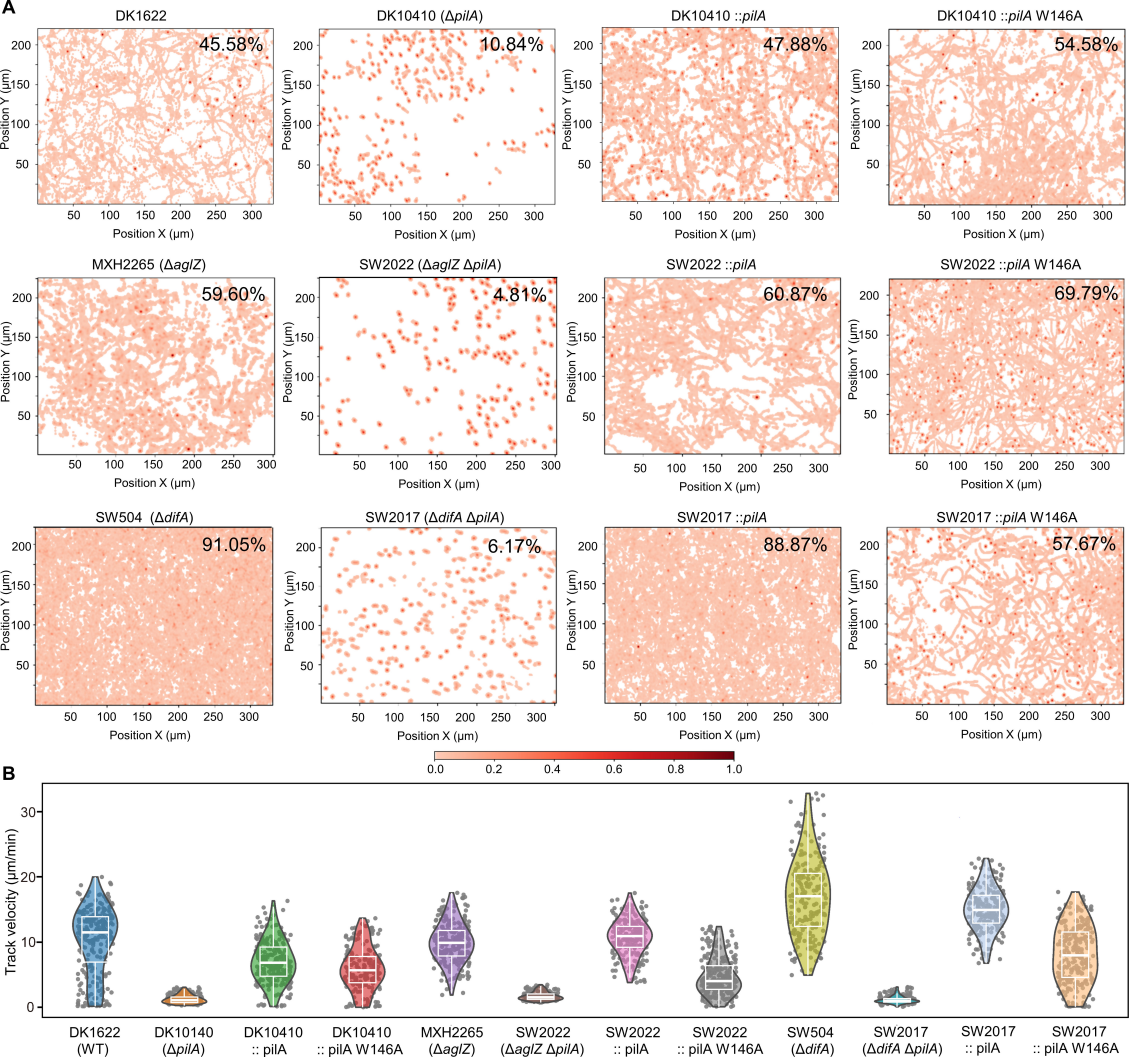
PilA-W146A enables enhanced surface exploration through EPS-independent T4aP retraction when submerged in a 1% methylcellulose solution. (**A**) Cell movements were recorded by time-lapse photography during a 1,000 s observation period. Cumulative surface coverage of the monitored cells is displayed. The red color indicates traversed (covered by bacterial trajectories) surface, and the color bar represents the detected bacterial density at each pixel point. The representative images are shown. (**B**) Statistical motion velocity distributions of the indicated strains, recorded from 1,000 s time-lapse tracking, are profiled by violin plots (μm/min).

To isolate the mechanical contributions of T4aP to cellular propulsion from EPS-dependent interactions, we utilized a 1% methylcellulose-supplemented liquid medium. This viscous environment allows T4aP anchoring to occur independently of the EPS matrix, enabling the analysis of single-cell S-motility (60, 61). WT cells exhibited surface motility (Fig. 8; Fig. S12) and occasional tethering to the surface via T4aP, resulting in an “upright position” (Fig. S12). This tethering behavior was absent in T4aP-deficient cells (DK10410), but retained in the W146A mutant, confirming the production of surface pili. To quantify surface exploration patterns, we employed a real-time tracking algorithm for automated and high-throughput data acquisition. As shown in Fig. 8A and Fig. S12, the motility history of all cells within the field of view was tracked, with unvisited surface areas represented in white and visited areas in red. Spatiotemporal analysis revealed striking differences in surface coverage. WT cells achieved ~46% surface coverage, whereas *ΔpilA* cells covered only ~11% over a 1,000 s observation period. Notably, the W146A mutant exhibited an enhanced exploratory phenotype, achieving ~55% surface coverage. Genetic epistasis analysis in SW2017 (Δ*difA*Δ*pilA*, EPS^-^, and PilA^-^) and SW2022 (Δ*aglZ*Δ*pilA*, A-motility^-^ and PilA^-^) backgrounds confirmed that this enhanced surface exploration was strictly T4aP-dependent (Fig. 8A). Paradoxically, instantaneous velocity profiling (Fig. 8B) showed that W146A cells moved at 6.02 ± 3.32 µm/min, compared with 10.36 ± 5.03 µm/min for WT cells (41.89% reduction, *P* < 0.001 by Student’s *t*-test). As previously reported, WT cells deposit residual EPS on solid surfaces during movement, which physically constrains trajectories of neighboring cells by T4aP-mediated recognition (45). Although WT cells exhibit faster S-motility rates, their capacity for surface exploration is limited by the EPS-T4aP interaction. Consistent with this, Δ*difA* mutants, lacking EPS production, exhibited significantly enhanced surface coverage (~91%). For the W146A mutant, despite reduced single-cell motility velocity compared with WT, the loss of EPS-binding capability in T4aP allows it to bypass EPS-mediated spatial constraints, thereby improving surface exploration efficiency. This is supported by the observation that SW2017::*pilA* W146A mutant exhibited ~58% surface coverage, which was statistically indistinguishable from the W146A mutant (*P* = 0.697, by Student’s *t*-test). Moreover, the combined effect of fewer functional pili (Fig. 7A) and slower retraction-driven motility (Fig. 8B) underlies the reduced surface coverage of the W146A mutant compared with the WT in the *ΔdifA* background.

### Aromatic residue conservation at PilA position 146 is crucial for T4aP-EPS interaction in *M. xanthus*

The C-terminal domain of PilA, which mediates surface adhesion and EPS interaction (62, 63), exhibits conserved structural motifs across species. Comparative analysis of 12 *Myxococcus* PilA homologs (Fig. S14) revealed a highly conserved N-terminal tail (98.4%–100% identity across residues 1–66) and a variable C-terminal region (50.6%–99.3% identity in residues 67–220). Notably, position 146 was predominantly occupied by tryptophan (8/12 species), with methionine substitutions observed in *M. xanthus* (WP_201421982.1, WP_140866013.1), *Myxococcus sp.* (WP_141254828.1), and *M. vastator* (WP_163784636.1). This evolutionary pattern suggests tolerance for specific non-conservative substitutions at this mechanistically critical site.

**Fig 9:**
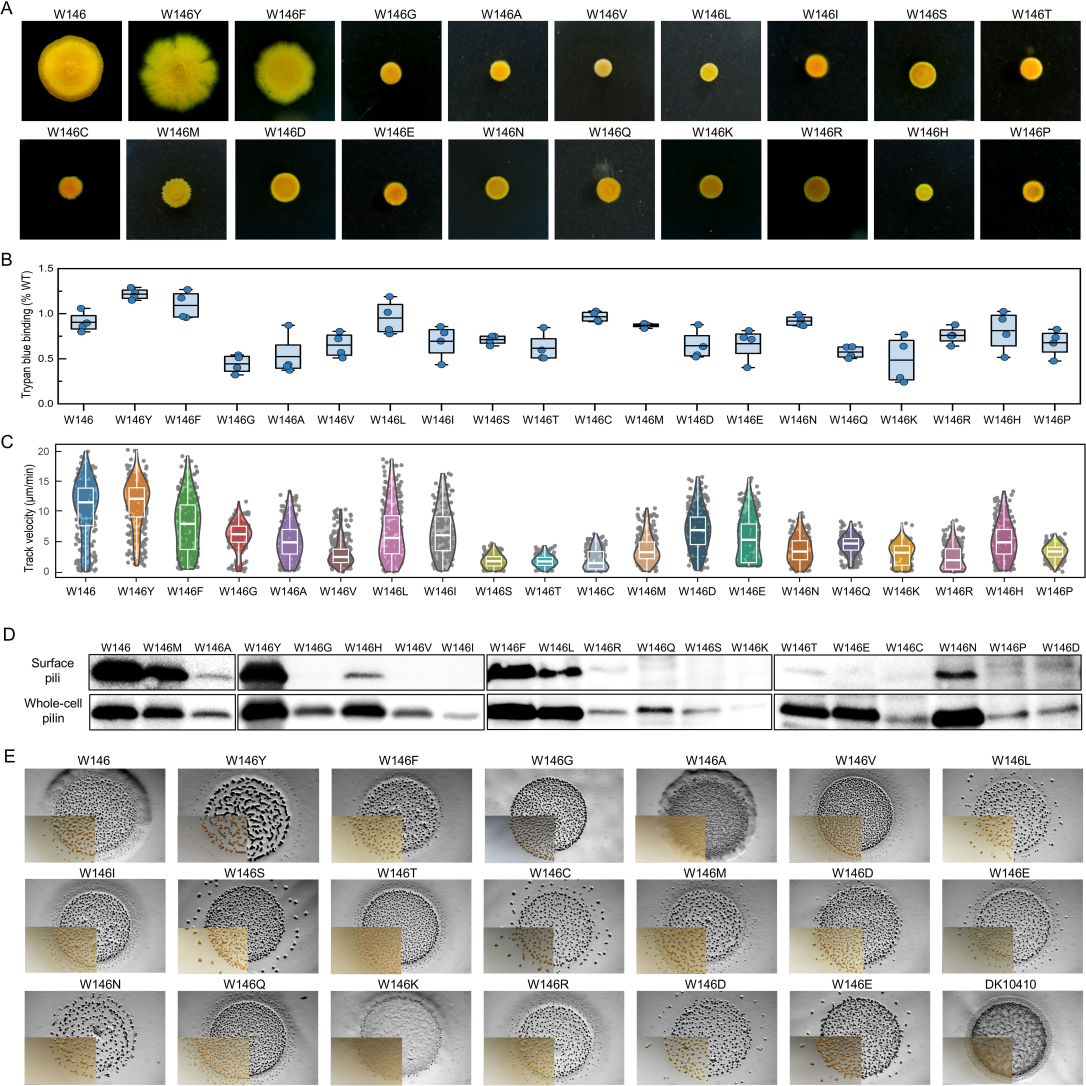
Phenotypic characteristics of *M. xanthus* cells harboring PilA mutants generated via saturation mutagenesis at the W146 site. (**A**) Colony expansion on 0.3% CTT agar after 5 days of incubation. (**B**) EPS production quantified via trypan blue binding assay. (**C**) Mean motility velocity of monitored cells in 1% methylcellulose solution, recorded over a 1,000 s period. (**D**) Surface-associated pili and whole-cell pilin levels in mutants, detected via western blot analysis. (**E**) Fruiting body formation of mutants after 72 h on 1.5% TPM agar, imaged using a stereo microscope.

Systematic saturation mutagenesis at W146 generated 19 variants, which were phenotypically characterized across five functional axes, that is, S-motility on agar, EPS production, single-cell S-motility, PilA biogenesis, and fruiting body development (Fig. 9). Substitution of tryptophan with other aromatic amino acids (W146Y/F) maintained WT-like S-motility on soft agar (Fig. 9A), EPS synthesis (Fig. 9B), single-cell movement (Fig. 9C), PilA production and T4aP assembly (Fig. 9D), and fruiting body formation (Fig. 9E). All mutants retained functional T4aP capable of extension/retraction, as evidenced by single-cell movement in methylcellulose medium, albeit with reduced average velocities compared to WT (Fig. 9C). In contrast, small aliphatic or polar substitutions (W146G/V/T/E) exhibited significantly reduced surface piliation, as confirmed by western blot (Fig. 9D). The W146M mutant displayed attenuated S-motility (reduced swarm diameter, rough colony edges) despite WT-level EPS, whole-cell pilin, and surface piliation. W146K substitution caused a marked reduction in whole-cell pilin, accompanied by diminished EPS production. Although most mutants retained normal developmental capacity, W146K failed to form fruiting body formation, and W146A produced immature structures (Fig. 9E). These findings demonstrate that aromatic residue conservation at position 146 is dispensable for T4aP biogenesis but critical for mediating EPS interactions. Non-aromatic substitutions disrupt piliation efficiency or EPS production, whereas methionine substitution specifically impairs S-motility, suggesting distinct mechanistic roles for this residue in pilus function and surface recognition.

## DISCUSSION

In *M. xanthus,* S-motility represents a sophisticated microbial locomotion system mechanistically analogous to twitching motility, both relying on T4aP dynamics (3, 5). Our current understanding posits that T4aP extends stochastically from the leading cell pole to engage with EPS coating on neighboring cells/substrata, thereby mediating coordinated group movement. Although prior studies have established EPS’s dual role in stimulating T4aP retraction and promoting multicellular pattern formation (15, 40, 44, 45), the molecular logic governing T4aP-EPS recognition remains enigmatic. This investigation sought to systematically address this knowledge gap through multimodal interrogation of the PilA-EPS interaction network.

Our quantitative binding analyses demonstrate that chitosan (partially deacetylated chitin) and isolated EPS exhibit specific affinity for PilA *in vitro*, corroborating earlier findings (15, 44, 45). Previous studies have identified significant levels of GlcNAc and GlcN residues in *M. xanthus* EPS (27, 29), and it has been proposed that monomeric GlcN and GlcNAc directly bind to PilA without inducing T4aP retraction (15). Notably, SPR and ITC experiments reveal selective GlcN-PilA interaction, contrasting with the undetectable GlcNAc binding under equivalent conditions. This specificity is supported by MD simulations, which delineate stable PilA-chitosan binding interfaces and differential interaction energies between GlcN and GlcNAc. The observed ambiguity in GlcNAc-PilA binding presents an intriguing paradox: although monomeric GlcNAc shows negligible affinity, polymeric chitin (a GlcNAc polymer) exhibits documented T4aP interaction (15). Our MD simulations suggest transient PilA-GlcNAc contacts may facilitate multivalent binding in polymeric contexts, potentially below detection thresholds of SPR and ITC. Although we identify GlcN as the key EPS monosaccharide mediating PilA interaction, several fundamental questions remain unresolved. For instance, how does the architecture of EPS, particularly the positioning of GlcN residues and the nature of glycosidic linkages, modulate PilA engagement? Spatial presentation of recognition motifs and cooperative binding effects may play critical roles in this interaction. These questions will be addressed once precise structural details of *M. xanthus* EPS are clarified.

To elucidate the structural basis of EPS sensing by PilA, we employed an integrative computational-experimental strategy that overcomes the throughput constraints of SPR. The predictive accuracy of our AlphaFold2 model was validated by comparing it with the recently published cryo-EM structure of PilA (23) and by recapitulating experimental binding phenotypes *in silico*. MD simulations and IFD calculations provided temporal resolution of binding dynamics, identifying tryptophan 146 (W146) as a critical aromatic residue within the globular C-terminal domain of PilA. Spatial mapping localized this residue in a solvent-exposed binding pocket predicted at the T4aP tip (Fig. S9B), positioning it ideally for initiating surface contact during motility. Mutagenesis studies confirmed the strict dependence of molecular recognition on W146. The PilACt-W146A mutation abolished GlcN/chitosan binding, whereas non-conservative mutations at Y69, W181, and F186 retained EPS affinity. Phenotypic assays revealed that PilA-W146A mutant cells produced retractable T4aP but exhibited impaired S-motility on soft agar. Notably, conservative substitutions (W146Y/F) preserved wild-type S-motility, underscoring the residue-specific role of W146 in carbohydrate recognition. This aligns with conserved mechanisms of carbohydrate-protein interactions, where aromatic stacking stabilizes sugar recognition (54). The spatial restriction of functional residues to position 146 suggests evolutionary optimization of T4aP architecture to prioritize rapid EPS engagement during pilus retraction cycles, thereby enhancing surface motility efficiency.

Our binding profiling revealed a striking specificity pattern: aminosugars (GlcN and ManN) exhibited significantly stronger affinity for PilACt compared with their N-acetylated derivatives. Initial hypotheses suggested electrostatic steering at physiological pH (7.6), where protonated GlcN (ζ-potential + 1.09 mV) could interact with the negatively charged surface of PilACt (ζ-potential −9.83 mV). Paradoxically, maximal binding occurred at pH 9.0, despite charge inversion (GlcN ζ −0.88 mV; PilACt ζ −10.73 mV), challenging simplistic electrostatic models. Consistent with these observations, IFD calculations of 3445 GlcN-PilA confirmations delineated a stereospecific recognition mechanism involving hydrogen-bond anchors, hydrophobic stabilization, and conformational selection. This hydrogen-bond network (Fig. 3G) rationalizes the pH-dependent affinity maximum: deprotonation of GlcN’s amine group (pKa ~7.58) enhances hydrogen-bonding capacity while retaining sufficient hydrophobicity for aromatic interactions.

Quantitative binding analyses further demonstrated that PilACt-carbohydrate interactions operate in an intermediate affinity regime. Apparent affinity constants (*K*_D_) are determined to be ~10^−4^ M for PilACt-polysaccharides and ~10^−5^–10^−4^ M for PilACt-monosaccharides using SPR. This moderate affinity contrasts sharply with canonical lectin-carbohydrate systems (*K*_D_ typically ~10^−6^–10^−9^ M) or enzyme-substrate recognition (*K*_D_ typically ~10^−4^–10^−10^ M) (64, 65) but aligns with the reversible binding nature of T4aP and EPS (12). The micromolar dissociation regime enables *M. xanthus* cells to rapidly form bonds during pilus extension, undergo force-dependent detachment at the end of retraction, and dynamically sample surfaces through stochastic binding events during S-motility. This balance of affinity and reversibility is critical for efficient surface exploration and motility.

The EPS trail-following behavior in *M. xanthus* exemplifies a sophisticated social navigation system where T4aP-EPS interactions serve dual roles: mechanical regulation, where EPS-mediated pilus retraction synchronizes multicellular movement; and spatial coordination, where EPS deposition creates chemotactic-like trails that guide collective migration. Alterations in T4aP specificity to EPS can lead to distinct physiological outcomes for *M. xanthus*. In methylcellulose-supplemented liquid medium, T4aP anchoring occurs independently of the EPS matrix, bypassing the retraction-stimulating function of EPS and enabling single-cell S-motility (60, 61). Complementation of the *pilA* gene in the PilA-deficient strain DK10410 did not fully restore its motility to the wild-type level. This difference might be attributed to the ectopic complementation of the *pilA* gene rather than an *in situ* restoration. For the W146A mutant, despite reduced single-cell motility velocity in methylcellulose medium compared with WT, the loss of EPS-binding capability allows T4aP to overcome EPS-mediated spatial constraints, thereby improving surface exploration efficiency. This creates a dynamic imbalance in the W146A mutant: preserved pilus retraction competence coupled with defective EPS recognition results in a unique motility paradigm. In this scenario, increased exploration efficiency masks underlying molecular defects in surface interaction regulation, underscoring the intricate interplay between T4aP mechanics and EPS-mediated spatial constraints. Notably, PilY1 and several minor pilins have been implicated in potential glycan-binding activity (66), adding a layer of complexity to the EPS trail-following behavior in *M. xanthus*. A systematic dissection of the respective roles and possible functional redundancy between these minor pilins and the major T4aP machinery in EPS recognition and collective motility is therefore essential. Moreover, the observation that T4aP-mediated anchoring can occur independently of EPS in methylcellulose medium underscores the critical influence of surface physicochemical conditions. This functional dichotomy highlights a key unanswered question: how does *M. xanthus* integrate these diverse strategies to selectively or cooperatively modulate T4aP-mediated motility in response to environmental cues?

The observed diversity at position 146 of PilA among *Myxococcus* strains suggests potential variations in EPS recognition capabilities. For example, in *M. vastator* (WP_163784636.1), residue 146 is methionine (M) rather than W. When this residue was introduced into *M. xanthus* DK1622 as a PilA-W146M mutant, the strain exhibited stable and retractable surface pili with detectable EPS levels. However, its motility on soft agar was impaired, indicating that changes in key residues like W146 may alter the specificity of DK1622’s EPS recognition, potentially enabling cross-recognition of EPS from other *Myxococcus* strains. As documented previously, the coexistence of distinct fruiting bodies of *Myxococcus* in the same niche highlights the ability of these strains to discriminate between individuals within mixed communities (67). Future research could explore the role of T4aP-EPS interactions in the isolation of multicellular structures through motility, potentially revealing mechanisms that govern social behavior and community organization in these bacteria. Furthermore, tryptophan residues are conserved in pilin proteins across diverse species, including *Escherichia coli*, *Geobacter sulfurreducens*, *N. gonorrhoeae*, *Neisseria meningitidis*, and *P. aeruginosa*, where they are consistently positioned within structurally analogous loop regions (23). This notable conservation underscores the critical functional importance of these aromatic residues, suggesting they constitute an evolutionarily maintained molecular interface that mediates T4aP interactions with extracellular components. In pathogenic species such as *N. gonorrhoeae*, *N. meningitidis*, and *P. aeruginosa*, this conserved interface is likely instrumental in supporting virulence, particularly during the initial stages of tissue attachment facilitated by T4aP. Consequently, targeting this aromatic residue site with small-molecule inhibitors presents a promising strategy to disrupt pilus-mediated functions across a broad spectrum of pathogens, thereby opening avenues for developing novel broad-spectrum anti-virulence therapeutics (68). The inherent flexibility of the loop region provides a dynamic and adaptable platform for substrate recognition and ligand binding. Allosteric binding of a molecule can induce conformational shifts in the loop, thereby modulating the protein’s activity by opening or closing the active site (69). The strategic placement of aromatic residues within these conformationally flexible loops enables the pilin protein to undergo substantial structural rearrangements. This molecular adaptability is critical for substrate envelopment, ensuring both tight and specific binding interactions.

The direct interaction between T4P and carbohydrates represents an evolutionarily conserved mechanism across diverse bacterial species, driving critical biological processes through species-specific adaptations. In *P. aeruginosa*, T4aP-mediated twitching motility is potentiated by the secretion of extracellular polysaccharides, which not only enhances pilus-surface adhesion forces but also establishes navigable polysaccharide trails for coordinated cellular migration (17, 70–72). Similarly, *Caldicellulosiruptor bescii* leverages its type IV pilin to directly bind plant-derived xylan and cellulose, conferring a competitive advantage in lignocellulose degradation ecosystems (73). Beyond environmental adaptation, *P. aeruginosa* employs conjugative T4bP to recognize D-rhamnose homopolymers in the lipopolysaccharides of recipient cells, initiating pathogenicity island transfer, showing a paradigm of glycan-mediated horizontal gene exchange (74). Pathogenic strategies further exemplify this mechanism, where *N. meningitidis* T4P specifically interacts with triantennary sialylated poly-N-acetyl lactosamine on human CD147/Basigin, enabling host-specific colonization through precise glycoconjugate recognition (75). These multifaceted examples, spanning surface motility, niche dominance, genetic exchange, and host-pathogen interplay, collectively underscore that T4P-carbohydrate interactions constitute a common molecular language governing some bacterial collective behaviors. Decoding this chemical-mechanical language will illuminate fundamental principles underlying surface sensing, biofilm architectonics, colonization dynamics, and virulence regulation. This insight not only advances our understanding of microbial ecology but also offers strategic avenues for manipulating bacterial communities and combating infectious diseases.

## MATERIALS AND METHODS

### Bacterial strains, plasmids, and growth conditions

The bacterial strains utilized in this study are detailed in FS1. All *M. xanthus* strains were routinely cultured at 30°C in liquid CTT medium (76) with shaking at 200 rpm or on CTT agar (1.5%) plates. All *E. coli* strains were grown at 37°C in LB broth (77). When applicable, kanamycin (Kan, 40  µg/ mL), ampicillin (Amp, 100  µg/mL), or apramycin (Apra, 20  µg/mL) was supplemented to the media for selection.

### Construction of *M. xanthus* mutants

Nonpolar in-frame deletion mutants were generated using a standard method (78). Upstream and downstream regions of the target gene were amplified and cloned into the *galK*-containing suicide plasmid pBJ113 (79) using the ClonExpress MultiS One Step Cloning Kit (Vazyme Biotech Co., Ltd., China). The deletion plasmids were electroporated into *M. xanthus* cells and integrated into the genome by single homologous recombination. The colonies that grew on CTT agar plates containing kanamycin were selected and inoculated onto CTT agar plates supplemented only with 1.0% D-galactose (Sigma-Aldrich, USA), where the unstable tandem duplication excised the plasmid by homologous recombination. The screened strains retain either the original WT locus or the deleted one, depending on where the recombination occurred. The obtained strains were further verified by colony PCR and sequencing.

Ectopic genetic complementation was performed using the vector pSWU19 (80), which contains an Mx8 *attP* locus, enabling integration at the chromosomal Mx8 *attB* site by site-specific recombination. Nucleotide sequences of the full-length *pilA* and its native promoter (300 bp upstream sequences) were PCR-amplified from the genomic DNA of *M. xanthus* DK1622 and inserted into the XbaI and EcoRI sites of pSWU19. Site-directed mutagenesis of PilA was introduced into pSWU-*pilA* using the Mut Express II Fast Mutagenesis Kit V2 (Vazyme Biotech Co., Ltd., China). After electroporation of the recombinant plasmids, the complementary strains were selected on CTT agar plates supplemented with kanamycin and further verified by colony PCR and sequencing.

The eGFP and mCherry genes were amplified and cloned into an autonomously replicating vector, pZJY4111 (81). The resulting plasmids were electroporated into the target strains. The fluorescence strains were cultured on CTT agar plates containing apramycin and were further verified by colony PCR and sequencing.

The primers and plasmids used in this study are listed in Table S1.

### Protein expression and purification

The reported vector pMXE01 (44) was used for the cloning and expression of truncated PilA (PilACt). Site-directed mutagenesis of PilACt was introduced into pMXE01 as described above. PilACt and its mutants (PilACt Y69A, PilACt W146A, and PilACt W181A) were expressed in *E. coli* BL21 (DE3). The cultures were grown in LB medium supplemented with ampicillin at 37°C to an OD_600_ value of 0.8 and induced with 0.1 mM IPTG and expressed at 16°C for 20 h. The cells were harvested and lysed in buffer (136.89 mM NaCl, 2.67 mM KCl, 8.1 mM Na_2_HPO_4_, 1.76 mM KH_2_PO_4_, pH 7.6). The lysate was loaded onto a HisTrap-HP column (Cytiva, USA) and washed with 20 column volumes of PBS buffer. The bound His-tagged protein was eluted with a linear gradient from 0 to 0.5 M imidazole in elution PBS buffer. Eluted proteins were concentrated and loaded onto a gel filtration column (HiLoad 16/600 Superdex 200  pg, Cytiva, USA). Peak fractions were pooled and concentrated to 2 mg/mL. The protein concentration was determined using the Bradford Protein Quantification Kit (Sangon Biotech, Co., Ltd., China).

### Isolation and purification of EPS

The insoluble EPS (I-EPS) and soluble EPS (S-EPS) of DK1622 cells were purified following the procedure as previously described (27, 33). The sample was incubated at 37 ^o^C with 200 µg/mL DNase I and RNase (Sigma-Aldrich, USA) for 24 h to remove remaining DNA and RNA contaminations. The Sevag assay was employed to remove the residual proteins (82). The carbohydrate content of purified EPS was determined using the anthrone assay (83).

### Fluorescent labeling of biofilms and planktonic cells

To probe for T4aP and EPS, DK1622 biofilms were allowed to form on the glass-bottom culture plates (NEST Biotechnology, Co., Ltd., China), following a previously described method (33, 84). The biofilms were then gently washed three times with PBS and blocked in PBS containing 3% (wt/vol) bovine serum albumin (BSA, Sangon Biotech, Co., Ltd., China). Next, the biofilms were incubated with rabbit polyclonal antibodies against PilACt (customized and produced by ABclonal Technology Co., Ltd., China, diluted 1: 5,000 in PBS buffer with 3% BSA) for 2 h at room temperature. The biofilms were washed three times with PBS and incubated with a 1:2,000 dilution of goat α-rabbit IgG conjugated to Alexa Fluor 647 (Abcam, United Kingdom) for 1 h at room temperature. After three additional washes with PBS, the biofilms were counterstained with Alexa 350-conjugated wheat germ agglutinin (WGA) and SYTO 9 (Thermo Fisher Scientific, USA). Planktonic cells were collected and fixed with 2% paraformaldehyde for 20 min. Subsequently, the cell pellet was harvested for fluorescent labeling by centrifugation at 4,000 × *g* for 5 min. The samples were observed under a LSM 900 confocal microscope (Zeiss, Germany), and images were rendered with Zeiss Zen software (version 2.6). Quantitative colocalization analysis of PilA and EPS in DK1622 biofilms was performed using colocalization colormap and JACoP plugin in ImageJ (85). Spatial correlation was evaluated using overlap coefficients M1 (PilA-EPS overlap) and M2 (EPS-PilA overlap), Pearson’s correlation coefficient (PCC), and intensity correlation quotient (ICQ), derived from fluorescence signals of PilA (red) and EPS (blue). The PCC and ICQ values measure the correlation of intensity distribution between channels, ranging from −1 (perfect negative correlation) to + 1 (perfect positive correlation) and from −0.5 (complete segregation) to 0.5 (complete colocalization), respectively (86). The M1 coefficient represents the proportion of the red signal overlapping with the blue signal, whereas M2 reflects the proportion of the blue signal overlapping with the red signal.

### Western blot analysis of whole-cell pilin and surface pili

Western blot analysis was performed as described previously (40). For whole-cell pilin, *M. xanthus* cells were lysed by ultrasonication, and the suspension was sedimented at 13,500 g for 5 min. The supernatant was transferred to a clean tube. For surface pili detection, T4aP were sheared from *M. xanthus* cells by vigorous vortexing for 20 min, followed by a 5 min centrifugation to remove the cell pellet. Pilin/pili in the solution were precipitated by adding MgCl_2_ to a final concentration of 100 mM. After incubating on ice overnight, the samples were centrifuged at 13,500 *× g* for 30 min at 4°C. The collected precipitate was resuspended in PBS buffer, mixed in equal volume with 2 × sample loading buffer (0.125 mol/L Tris-HCl, 4% SDS, 20% glycerol, 10% 2-mercaptoethanol, 0.002% bromophenol blue, pH 6.8), and boiled at 100°C for 10 min. Proteins were separated by SDS-PAGE and transferred to PVDF membranes. Blocking was performed using 3% BSA in TBST buffer (5 mM Tris-HCl, 150 mM NaCl, 0.05% Tween-20, pH 7.2) overnight at 4°C with gentle shaking. The anti-PilACt antibodies were used at a 1:5,000 dilution, followed by horseradish peroxidase-conjugated goat anti-rabbit antibodies (Sigma-Aldrich, USA) at a 1:5,000 dilution. Blots were developed using the Pierce ECL Western Blotting substrate (Cytiva, USA). Images were acquired using the ChemiDoc XRS system (Bio-Rad, USA).

### Pilus precipitation assay

The pilus precipitation assay was performed as previously described (33). The isolated pili/pilin and purified PilACt proteins (at a final concentration of 0.2 mg/mL) were incubated with either MOPS buffer, 0.5 mg/mL purified I-EPS, or 0.5 mg/mL chitin at 30°C for 1 h. The mixtures were pelleted by centrifugation at 13,000 *× g* for 10 min. The sediment was resuspended in 80 µL of 1% SDS, followed by boiling for SDS-PAGE and western-blot analysis.

### Surface plasmon resonance (SPR) analysis

The binding affinity of carbohydrates to PilACt and its mutants was determined by SPR measurements at 25°C using Biacore T200 (Cytiva, USA) in PBS buffer. The purified PilACt or its mutants (10 µg/mL in 10  mM sodium acetate, pH 4.5) were immobilized on the CM5 chips (Cytiva, USA) via amine coupling. Unreacted sites were blocked with 1.0 M ethanolamine-HCl (pH 8.5). Flow path 1 or path 3 was used as an activated blank control for subtraction. In all interaction experiments, analytes were injected at a flow rate of 30 µL/min for 180 s, with gradient concentrations programmed from low to high. After injection, dissociation was allowed to occur with the buffer flow for 300 s to facilitate chip regeneration. The resulting curves were corrected and fitted to the steady-state model using Biacore evaluation software (version 3.0, Cytiva, USA) to determine the dissociation constant (*K*_D_) values.

### Isothermal titration calorimetry (ITC) analysis

The calorimetric data for the binding of PilACt and its mutants to carbohydrates were measured at 25°C using a MicroCal PEAQ-ITC system (Malvern Instruments, United Kingdom). The carbohydrates, PilACt, and its mutants were dissolved in the PBS buffer. A 300  µL aliquot of protein solution was placed in the sample cell, followed by 19 sequential 2 µL injections of carbohydrates from a syringe stock solution into the sample cell. The injection interval was 150  s, and the agitation speed was set at 750  rpm. A control titration, in which carbohydrates were injected into PBS buffer under the same conditions, was performed to measure the heat of mixing and dilutions, which was then subtracted from the titration results involving the protein. Raw data were imported into MicroCal PEAQ-ITC analysis software (Version 1.1.01262) and corrected by subtracting the equivalent dilution heats of control titration. Integration of the area under each, along with deduction of the heat of dilution, yielded a thermogram of the molecular interaction between carbohydrates and PilACt or its mutants.

### Model building and refinement

A hybrid strategy integrating AlphaFold2 (multimer) and Rosetta was employed to predict and refine the *M. xanthus* T4aP structure. The 3D structure of PilA was predicted using AlphaFold2 (51, 87). Model quality was assessed using the PROCHECK program within SAVES (version 5.0, DOE-MBI Structure Lab UCLA). Structural visualizations were generated with PyMOL (version 2.4.1) (88).

The atomic modeling pipeline was initiated using the highest-confidence PilA monomer structure predicted by AlphaFold2. Model parameters were optimized according to CASP14 benchmarks, with the number of recycles set to 10, recycle early-stop tolerance at 0.5, and the number of ensembles set to 8.

During the AlphaFold-Multimer modeling process, the observed regular axial arrangement and fixed positional relationships between neighboring subunits informed the subsequent symmetric docking strategy. To generate right-handed helical assemblies, Rosetta symmetric docking was configured with the following parameters: a rise of 5–15 Å, a rotational angle of 80–130°, a radius to centroid distance of 15–30 Å, and 15 monomers per assembly. From 60,000 generated coarse-grained models, clustering (cutoff set as 1.0 Å) and energy scoring were performed, with root-mean-square deviation (RMSD) calculated against the regularly arranged subunit pairs. The model with the lowest energy was selected for high-resolution refinement.

To preserve the structural integrity of the pilus during refinement, a distance constraint was applied to the conserved F1/E5 pair (N-terminal Phe1 and Glu5), a known interaction that stabilizes T4P/T2SS architectures (89). From 10,000 high-resolution models generated, the pilus consistently adopted a single-start helical topology, completing one full rotation every 3–4 subunits, consistent with prior studies (20). Candidate models conforming to this geometry were visually screened, and those exhibiting atomic clashes were subsequently refined through molecular dynamics (MD) simulations, ultimately yielding an optimized T4aP structure for *M. xanthus* DK1622.

Finally, structural validation was carried out using the US-align tool, which computes Template Modeling (TM) scores on a 0–1 scale (where one indicates a perfect alignment). Residue-level alignments and TM scores were derived from these structural similarity metrics.

### Prediction of PilA binding pocket and binding sites

The PilA binding pocket was predicted by SiteMap implemented in Schrödinger (90), which employs Goodford’s GRID algorithm to identify energetically favorable interaction sites between molecular probes and the target protein (91). Blind docking experiments were performed via AutoDock Vina (92) to map potential ligand-binding sites on PilA. A total of 999 docking runs were executed using a grid box spacing of 126  × 126  × 126  Å^3^, encompassing the entire PilA structure.

### Molecular dynamics (MD) simulation

MD simulations of PilA-ligand complexes were performed using the AMBER force field (93). Specific force field parameters were assigned as follows: the Amber ff14SB for PilA (94), GLYCAM_06 j-1 for chitosan (95), and GAFF for monosaccharides (96). Missing parameters for ligand molecules were supplemented using the parmchk module in AmberTools.

Each prepared PilA-ligand complex was solvated in a cubic box of TIP3P water molecules (97), maintaining a minimum buffer distance of 15 Å from the solute. The system was neutralized by adding sodium counterions. The system preparation then proceeded through a series of steps: (i) energy minimization using a combination of steepest descent and conjugate gradient algorithms; (ii) gradual heating from 0 to 300 K over 60 ps with positional restraints applied to the solute; (iii) 1 ns of density equilibration under the NPT ensemble (constant number of particles, pressure, and temperature) using a Langevin thermostat and Berendsen barostat (98, 99); and (iv) an additional 1 ns of unrestrained NPT equilibration to ensure full temperature and pressure stabilization.

Production MD simulations were subsequently performed for 100 ns and 200 ns using GROMACS 2020.3 (100). The resulting trajectories were analyzed with built-in GROMACS utilities to calculate the root-mean-square deviation (RMSD) and root-mean-square fluctuation (RMSF). For the pilus structure refinement, both isolated monomers and assembled filaments underwent a separate 10 ns energy minimization procedure.

### Principal component analysis (PCA) and free energy landscape (FEL)

PCA was performed on Cα atoms of PilA using GROMACS tools, involving diagonalization of the covariance matrix to derive the first two eigenvectors (EVs), which capture dominant protein-ligand motion modes. FELs were constructed by estimating joint probability distributions across the essential subspace defined by the top two EVs.

### MM-PBSA calculations and per-residue free energy decomposition

Binding free energies were calculated using the molecular mechanics Poisson-Boltzmann surface area (MM-PBSA) method (101) implemented in AMBER software. Snapshots extracted from explicit-solvent MD trajectories were stripped of solvent molecules to compute molecular mechanics energies. Binding energy calculations were performed using AMBER and gmx_MMPBSA (102), with per-residue contributions decomposed to estimate individual amino acid involvement (103).

### Induced fit docking (IFD) analysis

GlcN was docked into the PilA binding pocket using Induced Fit Docking (IFD) in the Schrödinger suite. PilA’s structure was energy-minimized with the OPLS4 force field (104) using an implicit solvent model to retain native conformation while resolving steric clashes. GlcN’s 3D structure was generated via Schrödinger’s LigPrep module, and docking was performed in standard precision (SP) mode. Resultant interactions were visualized in PyMOL, with hydrogen bonds, hydrophobic contacts, and electrostatic forces mapped using LigPlot.

### Conservative analysis of PilA

The *M. xanthus* DK1622 PilA sequence (WP_011555734.1) was retrieved from NCBI (http://www.ncbi.nlm.nih.gov) and subjected to BLASTP against the *Myxococcus* (taxid:32) refseq_protein database. Homologs with > 50% identity/query coverage were aligned using MAFFT7 (105) and used to construct a neighbor-joining phylogenetic tree in MEGA7 (106). Sequence conservation was analyzed via ConSurf (Ashkenazy et al. 2016) using the predicted PilA structure and multiple sequence alignment.

### Zeta potential measurement

GlcN and PilACt were diluted in PBS buffer, and the solutions were adjusted to a suitable pH (6, 7.6, and 9) using either HCl or NaOH. The zeta (ζ) potential of GlcN (0.5 mg/mL) and PilACt (1 mg/mL) solutions was measured using UV-grade cuvettes with a Zetasizer NanoZS90 (Malvern Instruments, United Kingdom). For each measurement, 1 mL of the sample was injected into a folded capillary cell. All experiments were conducted in triplicate.

### T4aP-dependent motility assays

*M. xanthus* cells were grown in CTT medium for 20 h to the exponential growth phase and resuspended in TPM buffer (10  mM Tris-HCl, 1  mM K_2_PO_4_, 8  mM MgSO_4_, pH 7.6) to a calculated density of 5 × 10^9^ cells/mL. An aliquot of 2.5 µL concentrated culture was spotted onto CTT 0.3% agar plates to assess S-motility. When needed, purified I-EPS was gently and evenly coated on the surface of 0.3% agar in advance. After incubating for 5 days at 30°C, colony morphologies were captured using a 20 × microscope objective (Nikon Eclipse Ni, Japan) and a MetaMorph (version 7.04, Molecular Devices, USA) controlled camera.

### EPS quantification assays

EPS production was visualized using calcium fluorescent white (CFW, Sigma-Aldrich, USA), Congo red, and trypan blue (Sangon Biotech, Co., Ltd., China) binding assays (107, 108). Briefly, 2.5 µL of a cell suspension (5 × 10^9^ cells/mL in TPM buffer) was spotted onto CTT 0.3% agar plates containing CFW (50  µg/mL) or Congo red (30 µg/mL). After incubation at 30°C in the dark for 5 days, CFW fluorescence images were captured under exposure to a long-wavelength (365 nm) UV lamp using a handheld digital camera (Nikon, Japan). For the trypan blue binding assay, cultures were concentrated to 5 × 10^8^ cells/mL in TPM buffer. An aliquot of 125 µl of the cell suspension was mixed with the same volume of trypan blue stock solution (20 µg/mL). The TPM buffer mixed with trypan blue was used as a control. All samples were vortexed briefly and incubated for 30  min at room temperature in the dark. After centrifugation at 15,000 *× g* for 10 min, the supernatant was transferred, and the OD value was measured at 585  nm. The relative amount of trypan blue binding for each strain was normalized to that of the WT strain, which was arbitrarily set as 100%. Triplicate experiments were performed.

### Single-cell motility assays

The T4aP-mediated single-cell motility of *M. xanthus* was assayed as previously described (45). Briefly, the harvested *M. xanthus* cells were diluted in MOPS buffer (10  mM MOPS, 8  mM MgSO_4_, pH 7.6) to 5  ×  10^6^  cell/mL, and 5  µL of an exponentially growing cell culture was spotted on a 24-well polystyrene plate (NEST Biotechnology, Co., Ltd., China) at room temperature for 10  min in the dark to allow cells to settle on the bottom of the well. The cells were overlaid with 400  µL of MOPS buffer containing 1% methylcellulose and incubated in the dark at room temperature for 1 h before filming. Individual cell movements were captured by an inverted microscope (Nikon Eclipse TE200, Japan) with a 40 × objective and analyzed using MetaMorph software. Cell movement was recorded over a period of 1,000 s, with intervals of 10 s. An integrated system was developed that combines object recognition based on the Mask Region-based Convolutional Neural Network (R-CNN) architecture with a tracking system utilizing Graph Neural Networks (GNN). Within this system, individual bacterial entities were successfully identified and tracked, with positional and morphological data being output for subsequent analysis. To quantify overall surface coverage, each bacterium was approximated as an ellipse with the short-axis length used as the radius. For the visualization of spatial distribution, density heatmaps were generated. These heatmaps were based on a Gaussian-smoothed 2D histogram of cell center coordinates, where a color gradient was employed to represent regions of bacterial aggregation.

### Strain-mixing assays

The cells expressing eGFP or mCherry from exponentially growing cultures were mixed at a 1:1 ratio to a final concentration of OD_600_ 35. Two µL of mixed cultures were then spotted onto CTT 0.3% agar. After incubating at 30°C for 24 h, the swarming plates were photographed with an SMZ1500 stereomicroscope (Nikon, Japan), and the fluorescence microscopy imaging of swarms was captured using a 4 × or 20 × microscope objective (Nikon Eclipse Ni, Japan) and a MetaMorph (version 7.04, Molecular Devices, USA) controlled camera.

### Fruiting body formation assay

*M. xanthus* cells were grown in CTT to the exponential growth phase and concentrated to an OD_600_ of 10 in TPM buffer. Aliquots (5 µL) of concentrated cells were spotted onto CFL agar and incubated for 5 days at 30°C. Pictures of fruiting bodies were taken using a Nikon SMZ1500 stereomicroscope.

## SUPPLEMENTAL MATERIAL

Supplemental material mbio.02568-25-s0001.docx Supplemental figures and tables.

Movie S1 mbio.02568-25-s0002.mp4 MD simulation of the PilA-glucosamine complex.

Movie S2 mbio.02568-25-s0003.mp4 MD simulation of the PilA-glucose complex.

Movie S3 mbio.02568-25-s0004.mp4 MD simulation of the PilA-N-acetylglucosamine complex.

Movie S4 mbio.02568-25-s0005.mp4 MD simulation of the PilA W146A-glucosamine complex.

ASM does not own the copyrights to Supplemental Material that may be linked to, or accessed through, an article. The authors have granted ASM a non-exclusive, world-wide license to publish the Supplemental Material files. Please contact the corresponding author directly for reuse.
