# Supplementary material for: Aromatic residue Trp146 mediates dynamic interplay between type IVa pili and exopolysaccharide in social motility of Myxococcus xanthus
Source: mBio. 2025 Nov 17;16(12):e02568-25. doi: 10.1128/mbio.02568-25 (PMC12691611; doi:10.1128/mbio.02568-25)
Supplement: Supplemental material — Supplemental figures and tables. [file mbio.02568-25-s0001.docx]

**Supplementary information**

Aromatic residue Trp146 mediates dynamic interplay between type IVa pili and exopolysaccharide in social motility of *Myxococcus xanthus*

Yan Wang1¶, Yipeng Wang1¶, Weiwei Xue1¶, Jiaxin Li1, Fujian Zhang1, Xiashi Lv2, Fengyu Zhang1, Yuezhong Li1, Chuandong Wang1*, Wei Hu1*

1 State Key Laboratory of Microbial Technology, Microbial Technology Institute, Shandong University, Qingdao, Shandong, China

2 State Key Laboratory of Natural Medicines, Center of Drug Discovery, China Pharmaceutical University, Nanjing, China

***** Corresponding author

E-mail: hw_1@sdu.edu.cn (WH), wangchuandong@sdu.edu.cn (CW)

¶ These authors contributed equally to this work.

**
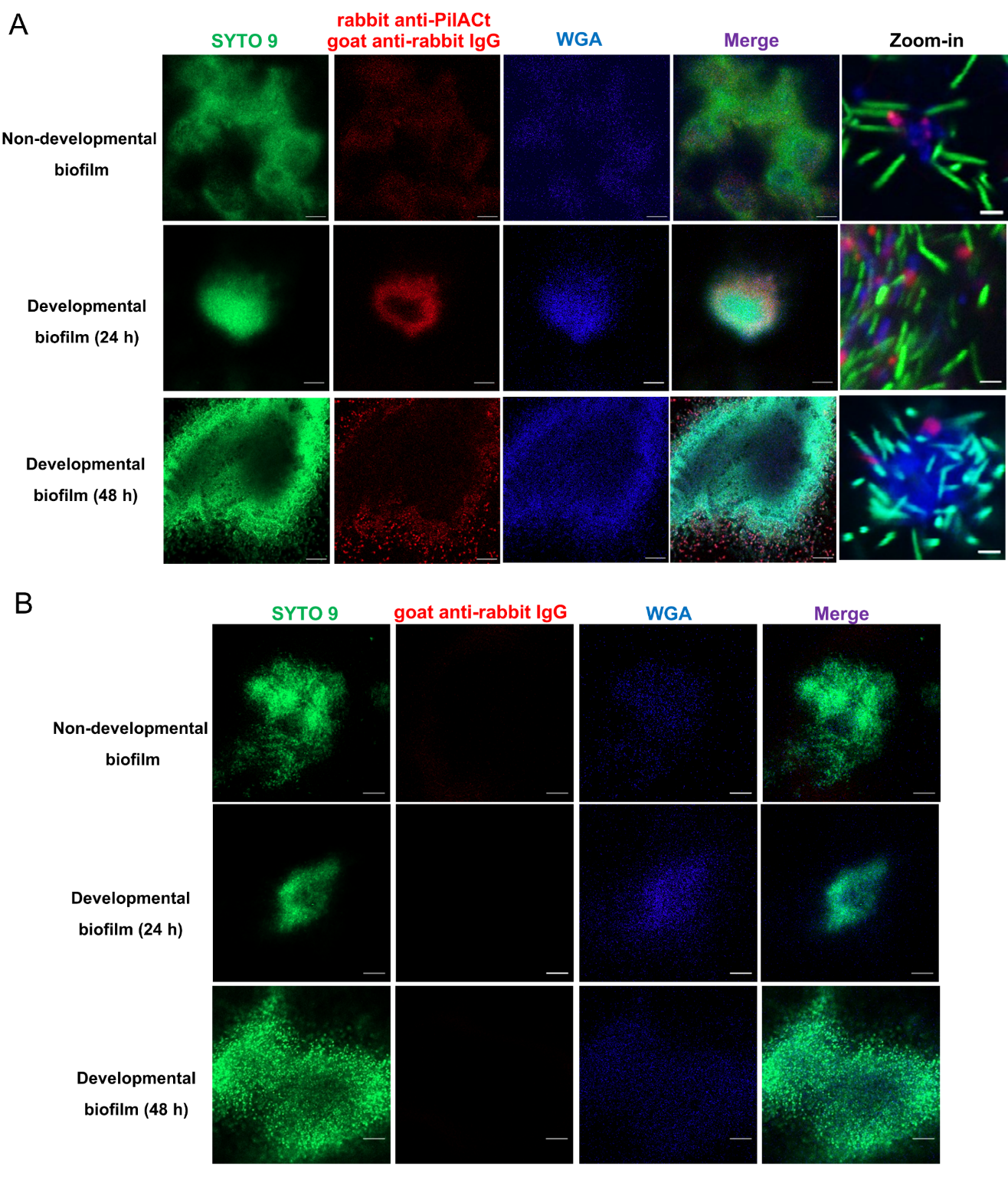
**

**Fig S1. Spatial organization of *M. xanthus* DK1622 biofilm components visualized by triple-channel fluorescence microscopy.** (A) *In situ* immunolocalization of PilA in DK1622 biofilms: Cells stained with SYTO 9 (green), EPS labeled with Alexa Fluor 350-WGA (blue), and PilA detected via sequential incubation with rabbit anti-PilACt polyclonal antibodies and Alexa Fluor 647-conjugated goat anti-rabbit IgG (red). (B) Negative controls processed identically without primary antibodies. Overview images of DK1622 biofilms were acquired using confocal laser scanning microscopy (CLSM) at a 40× magnification and zoom-in panels were acquired at a 60× magnification. Data shown are representative of four independent biological replicates. Scale bars: 20 μm (overview), 2 μm (zoom-in panels).


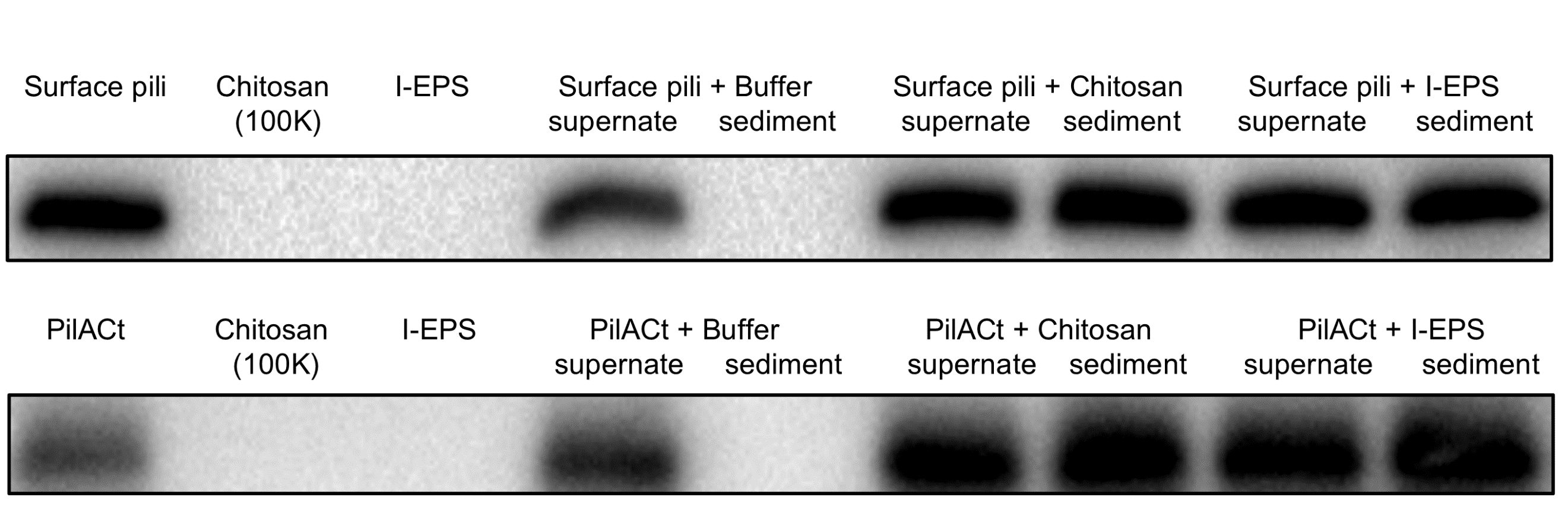


**Fig S2. Co-precipitation analysis validating PilA-EPS interaction.** Western-blot detection of *M. xanthus* DK1622 surface-sheared T4aP ("surface pili") and recombinant PilACt co-precipitated with insoluble EPS (I-EPS). Insoluble chitosan (100 kDa) served as positive control. Anti-PilACt antibodies (1:5,000 dilution) were used for immunodetection.


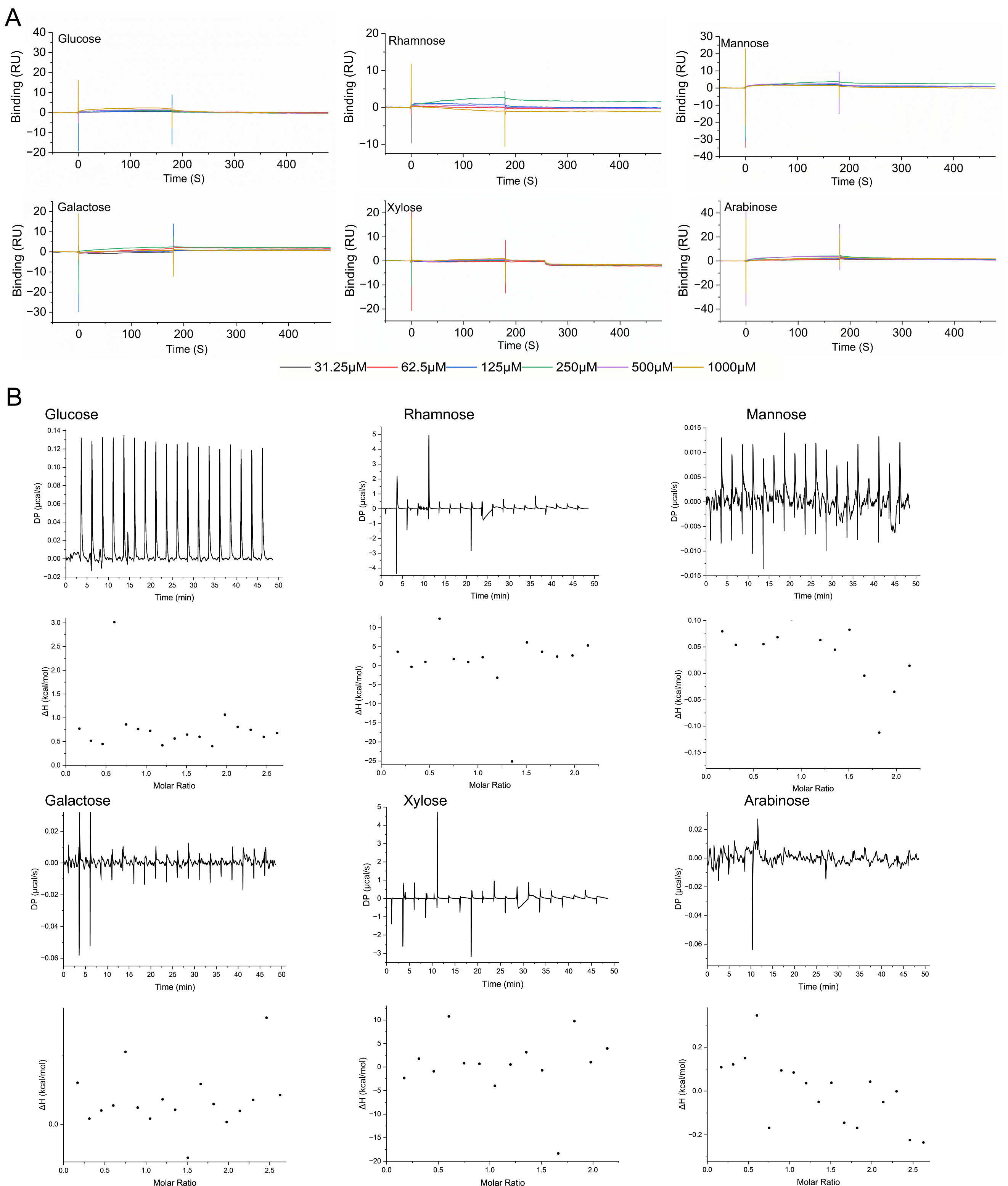


**Fig S3. Binding analysis of unmodified monosaccharides with PilACt by SPR and ITC.** (A) SPR sensorgrams. PilACt was exposed to gradient concentrations of glucose, rhamnose, mannose, galactose, xylose, and arabinose, respectively. RU: response units. (B) ITC thermodynamic profiles. Top: Raw heat changes during monosaccharide titration into PilACt. Bottom: Normalized enthalpy changes.

**
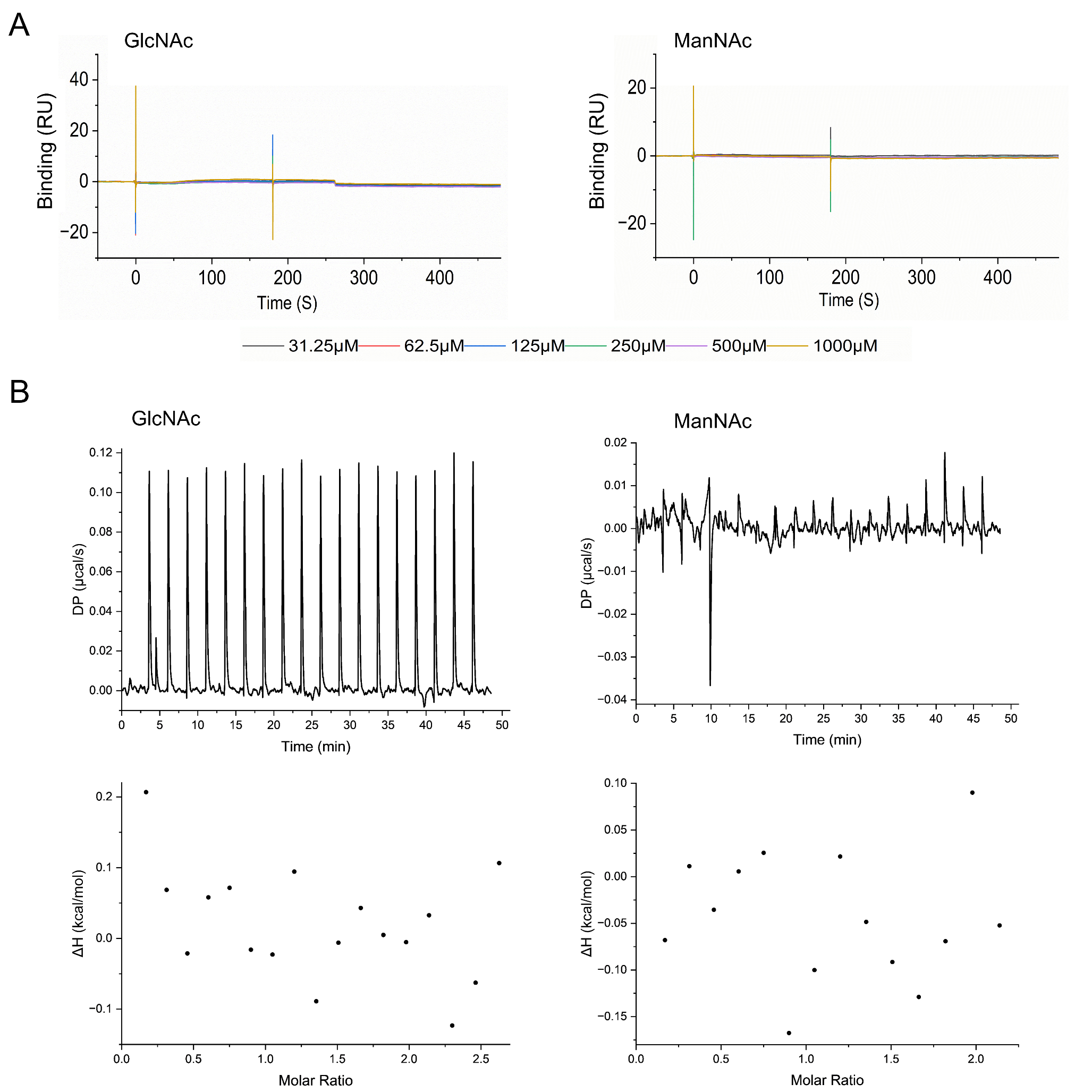
**

**Fig S4. Binding analysis of GlcNAc and ManNAc with PilACt by SPR and ITC.** (A) SPR sensorgrams. PilACt was exposed to gradient concentrations of GlcNAc and ManNAc, respectively. RU: response units. (B) ITC thermodynamic profiles. Top: Raw heat changes during monosaccharide titration into PilACt. Bottom: Normalized enthalpy changes.

**
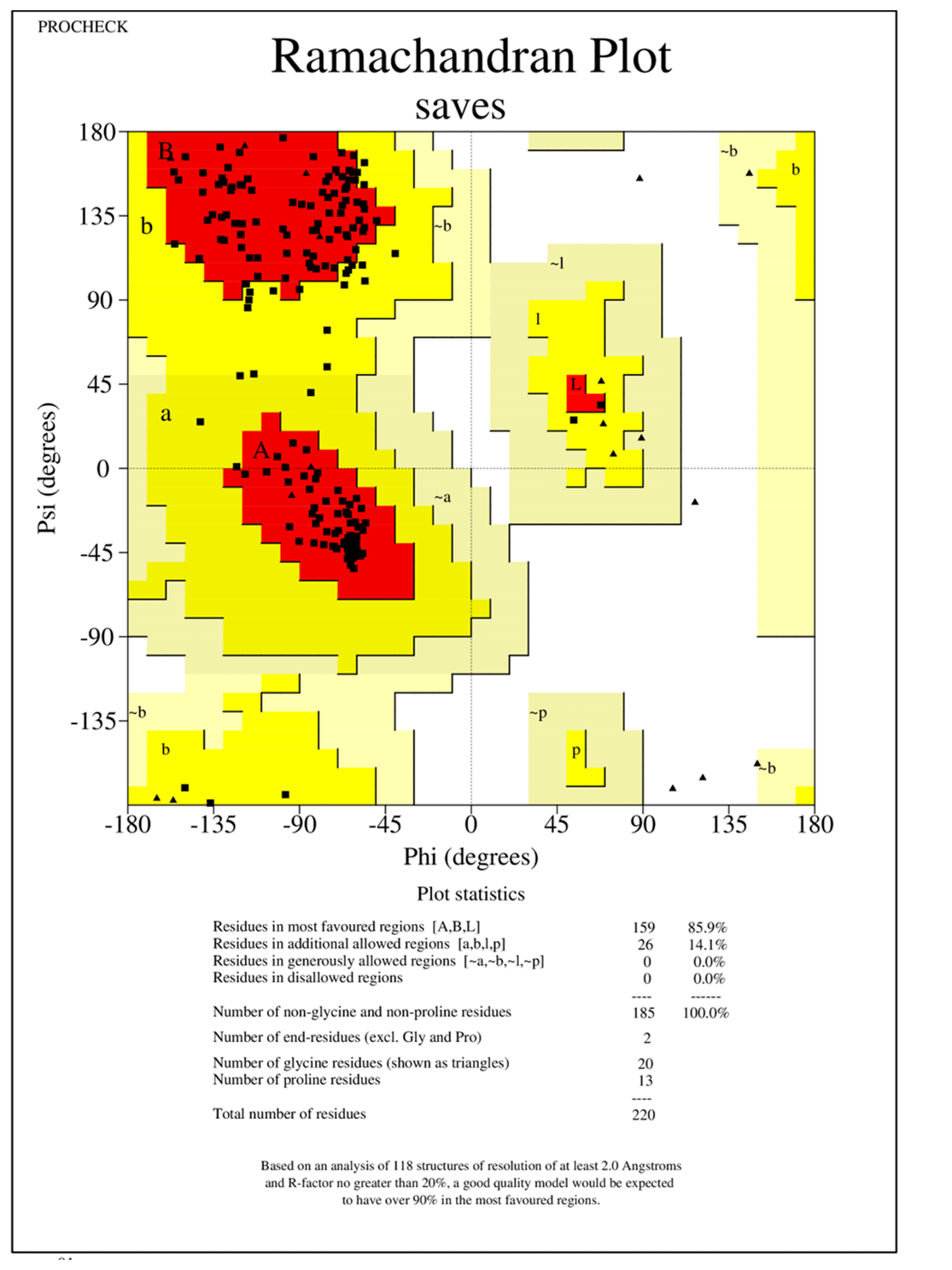
**

**Fig S5. Structural validation of *M. xanthus* PilA model through Ramachandran plot analysis.** PROCHECK-generated Ramachandran plot evaluates backbone dihedral angle distributions of the predicted PilA structure. All atoms were included in the analysis. Residues in the most favoured region are highlighted in red, while those in the additionally allowed region are shown in yellow. Residues in the generously allowed region are designated by a light yellow color. Residues located in the disallowed region are shown in white.


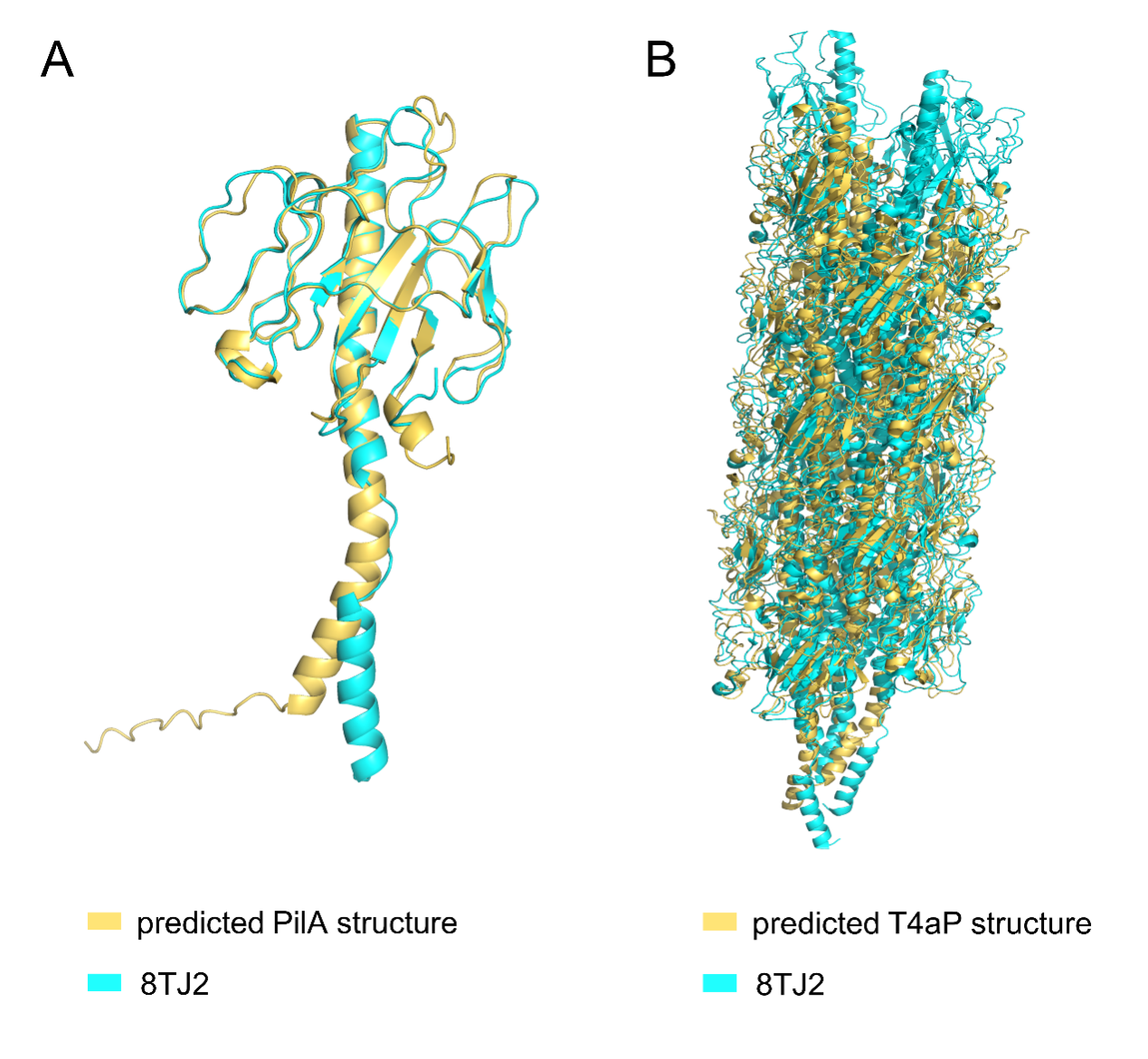


**Fig S6. Structural congruence analysis between predicted and experimental T4aP assemblies.** (A) PilA monomer alignment. TM-align comparison of AlphaFold2-predicted PilA (yellow) with the experimentally resolved structure from *M. xanthus* T4aP (PDB 8TJ2, cyan), and TM-score is 0.912 (0-1 scale, >0.5 indicates same fold). (B) Pilus filament comparison. Predicted T4aP model (yellow) aligned with cryo-EM structure (cyan, PDB 8TJ2), and TM-score is 0.917.


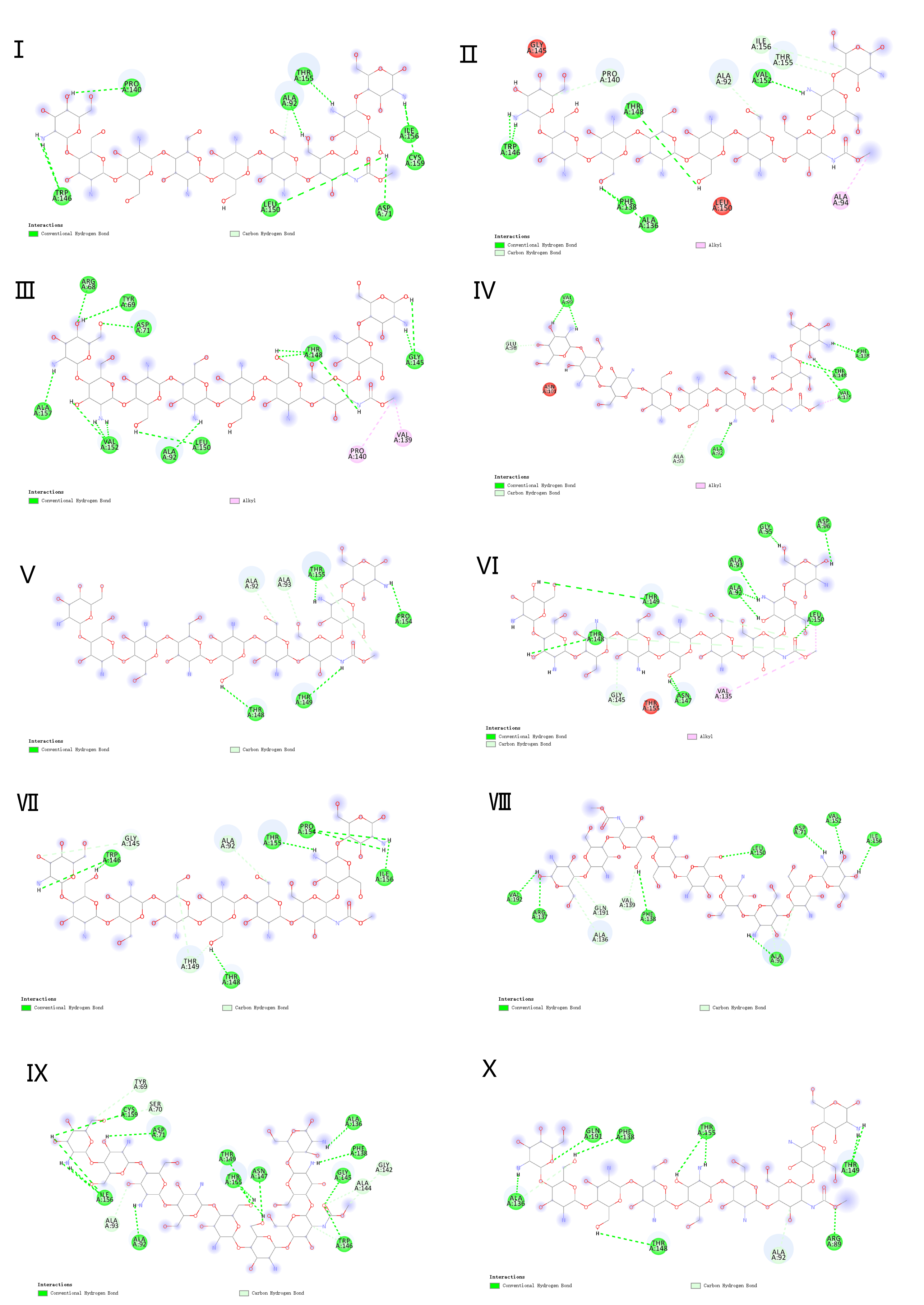


**Fig S7. Energetic ranking of PilA-chitosan interaction modes.** Top ten 2D binding poses predicted by AutoDock Vina, sorted by binding energy. Hydrogen and alkyl bond interactions are indicated by green and pink dashed lines, respectively.


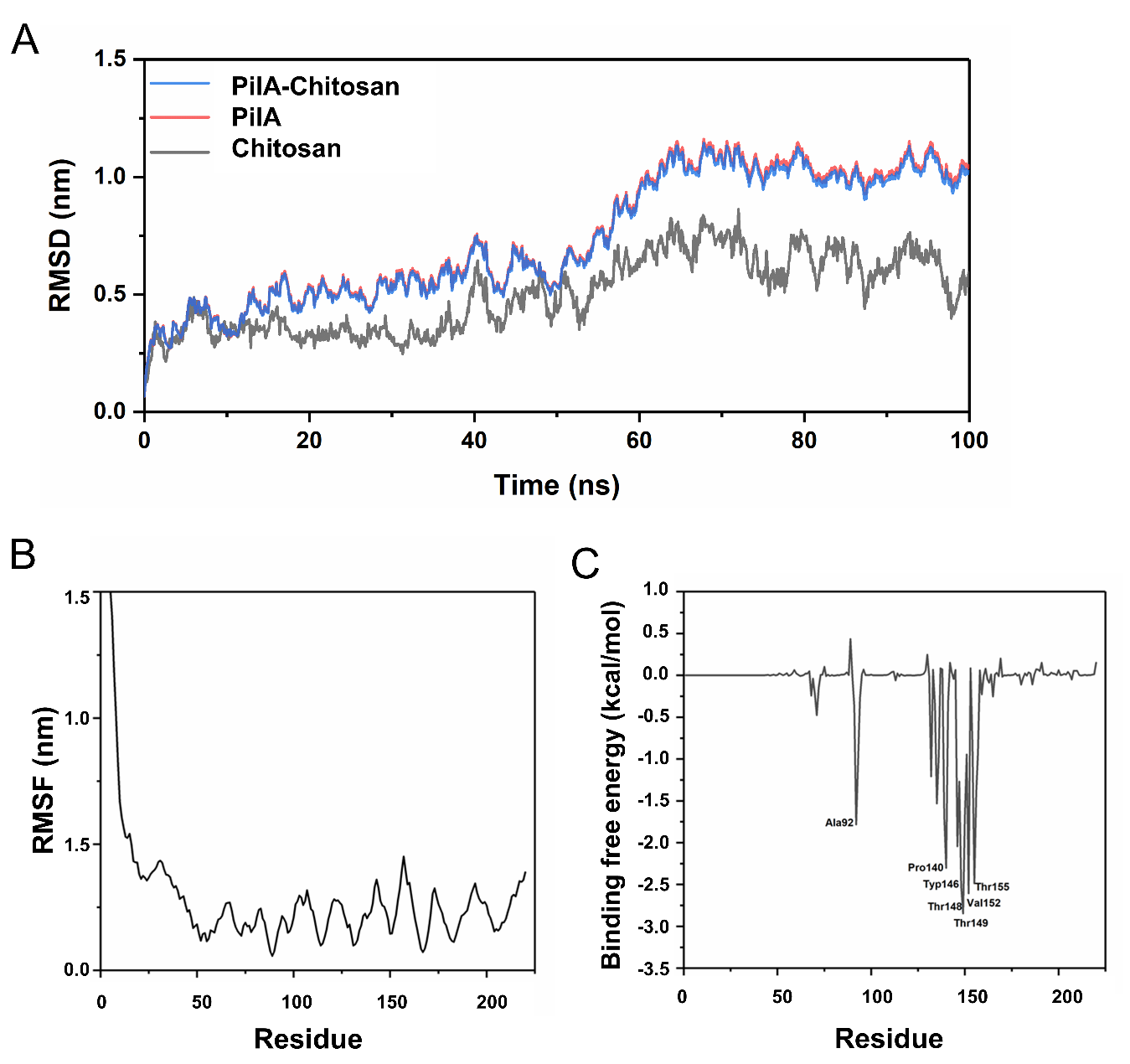


**Fig S8. Molecular dynamics characterization of PilA-chitosan binding.** (A) Root-mean-square deviation (RMSD) trajectories over 100 ns simulations for PilA (red), chitosan (grey), and PilA-chitosan complex (blue). (B) Residue flexibility profile (Root Mean Square Fluctuation, RMSF) of PilA residues. (C) Residue-wise decomposition of the binding energy of PilA-chitosan complex during MD simulation. The plot highlights the residues that have a high contribution to the total binding energy.

**
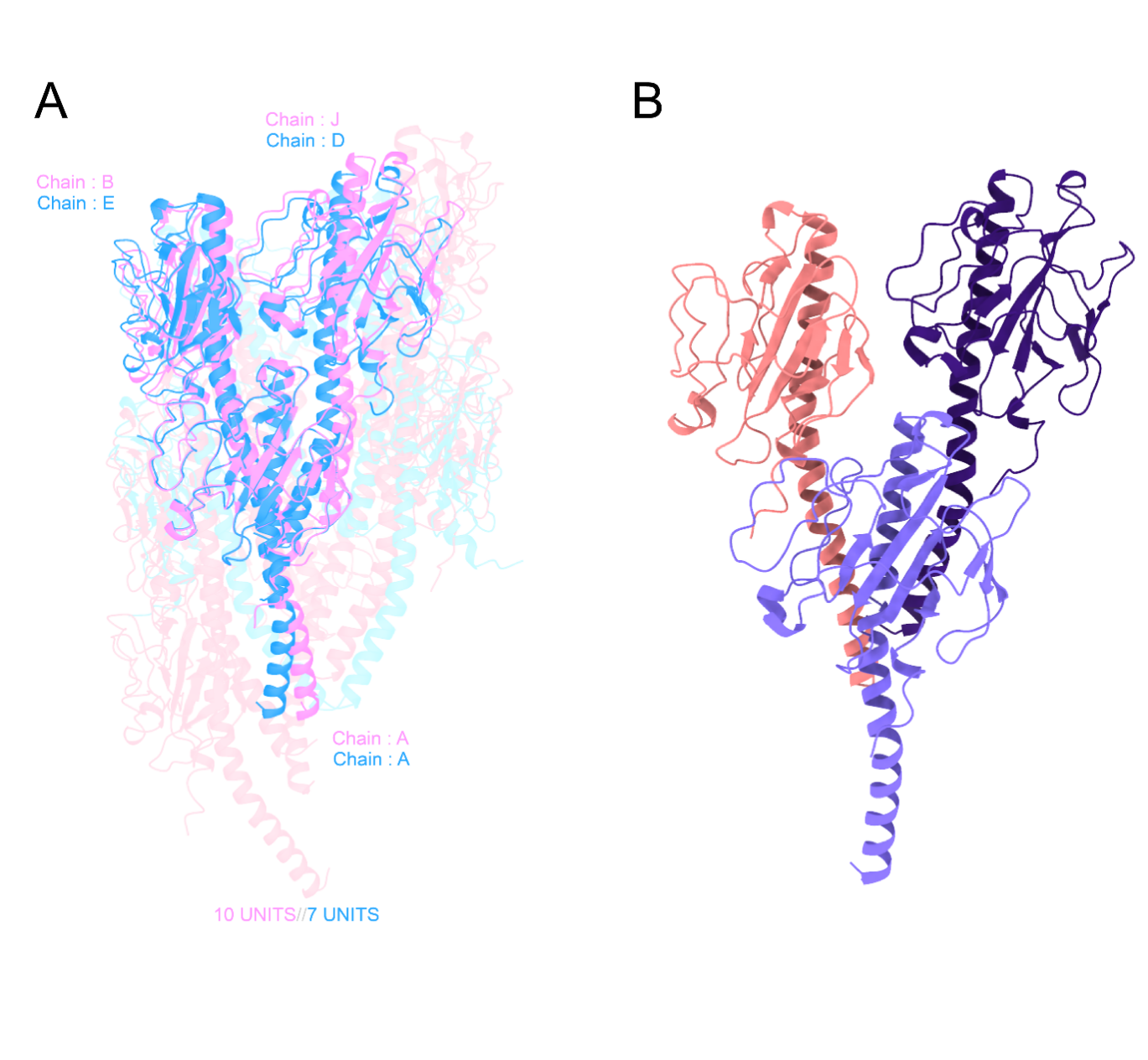
**

**Fig S9. Local consistency in PilA subunit interactions provides key constraints for pilus assembly prediction.** (A) Superimposition of PilA oligomer models generated by AlphaFold-Multimer, showing a 7-subunit assembly (PilA-7, various shades of blue/cyan) and a 10-subunit assembly (PilA-10, various shades of pink/magenta). Despite overall structural variations, a conserved local arrangement is evident. Specifically, the relative positions of three key side chains within interacting subunit triplets (e.g., Chain A, Chain D, and Chain E in PilA-7, analogous to Chain A, Chain B, and Chain J in PilA-10, based on the image labels showing Chain A [bottom], Chain B/E [middle], Chain D/J [top] as examples of interacting units) show high consistency between the PilA-7 and PilA-10 models. (B) Isolated view of two interacting PilA subunit triplets, highlighting the consistent spatial arrangement of the three interacting subunits and their conserved side-chain orientations (not explicitly shown but implied by the consistent backbone) that were observed.

**
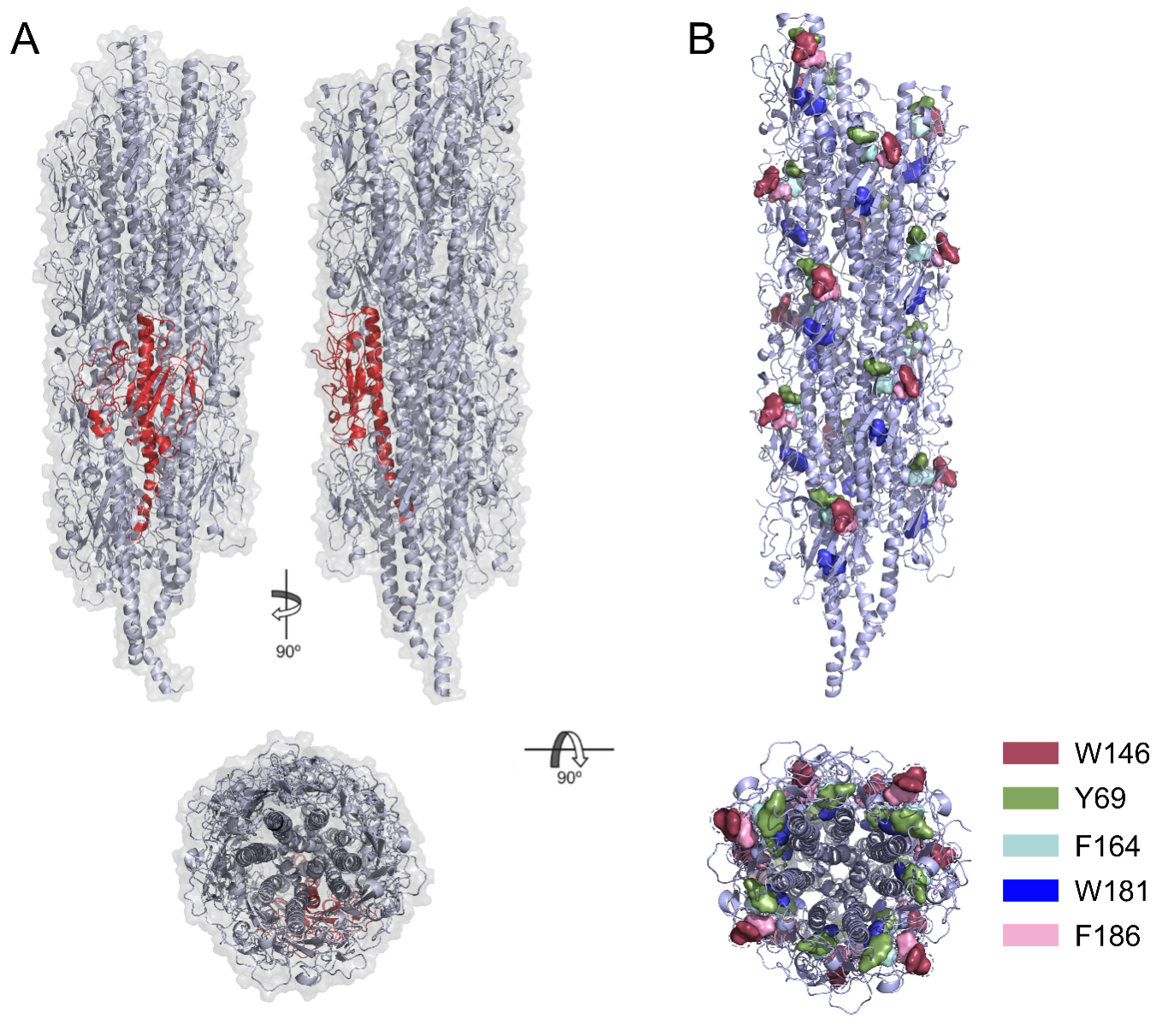
**

**Fig S10. Predicted structure of *M. xanthus* T4aP.** (A) Predicted T4aP architecture. Cross-sectional view showing helical arrangement of PilA subunits (red) within pilus shaft (grey surface, 80% transparency). (B) Functional hotspot mapping. Surface representation highlighting candidate binding sites on the predicted T4aP structure.


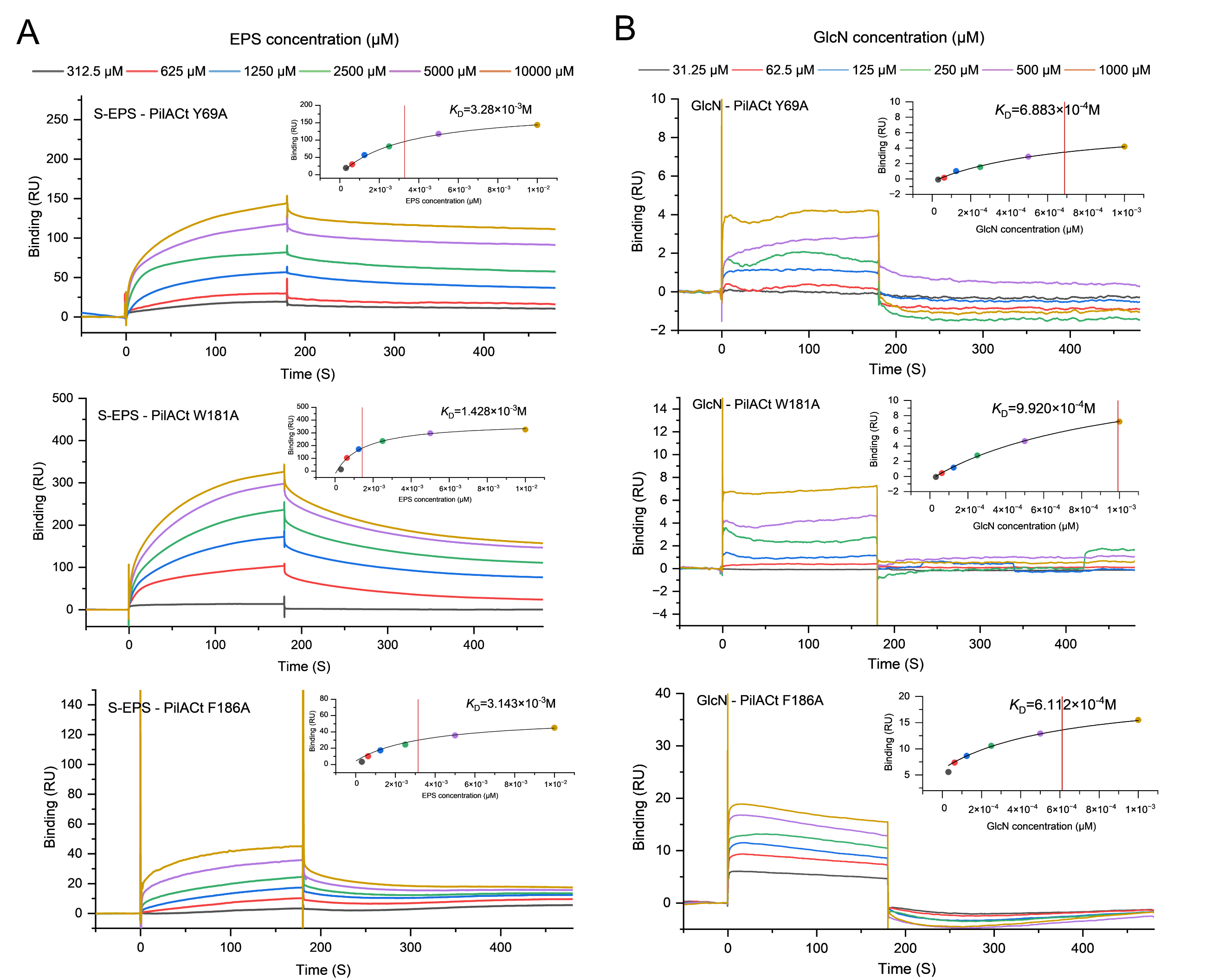


**Fig S11. Aromatic residue mutagenesis reveals W146 specificity in PilA-EPS recognition.** The binding affinity of single-residue variants PilACt-Y69A, PilACt-W181A, and PilACt-F186A to S-EPS (A) and GlcN (B) was evaluated by SPR analysis. Representative sensorgram and calculated *K_D_* values are shown. Color-coded lines indicate analyte concentrations. RU: Resonance units.


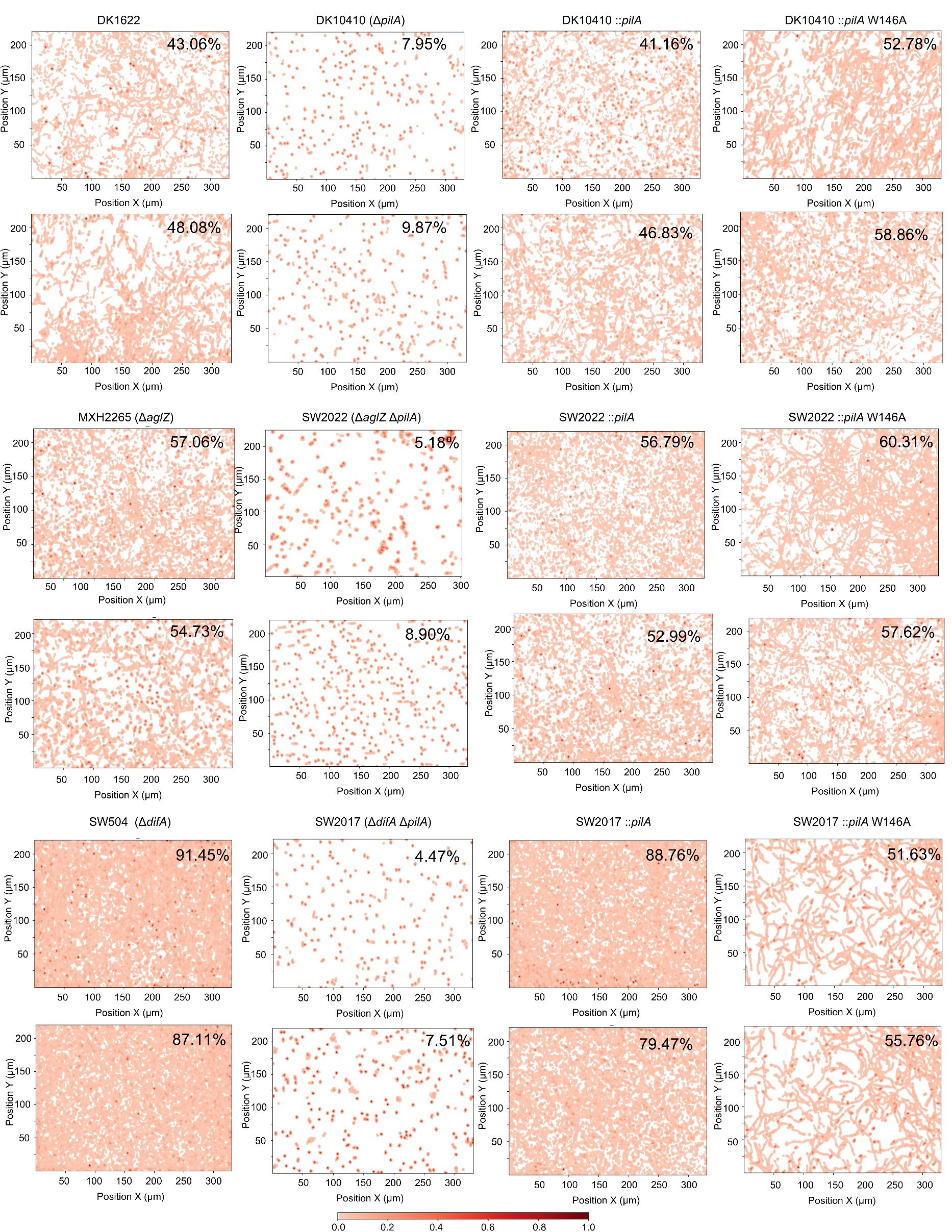


**Fig S12. Two additional experimental replicates of EPS-independent motility assays for the wild-type and mutant strains.** Cell movements were recorded by time-lapse photography during a 1000-second observation period. Cumulative surface coverage of the monitored cells is displayed. The red color indicates traversed (covered by bacterial trajectories) surface, and the color bar represents the detected bacterial density at each pixel point.

**
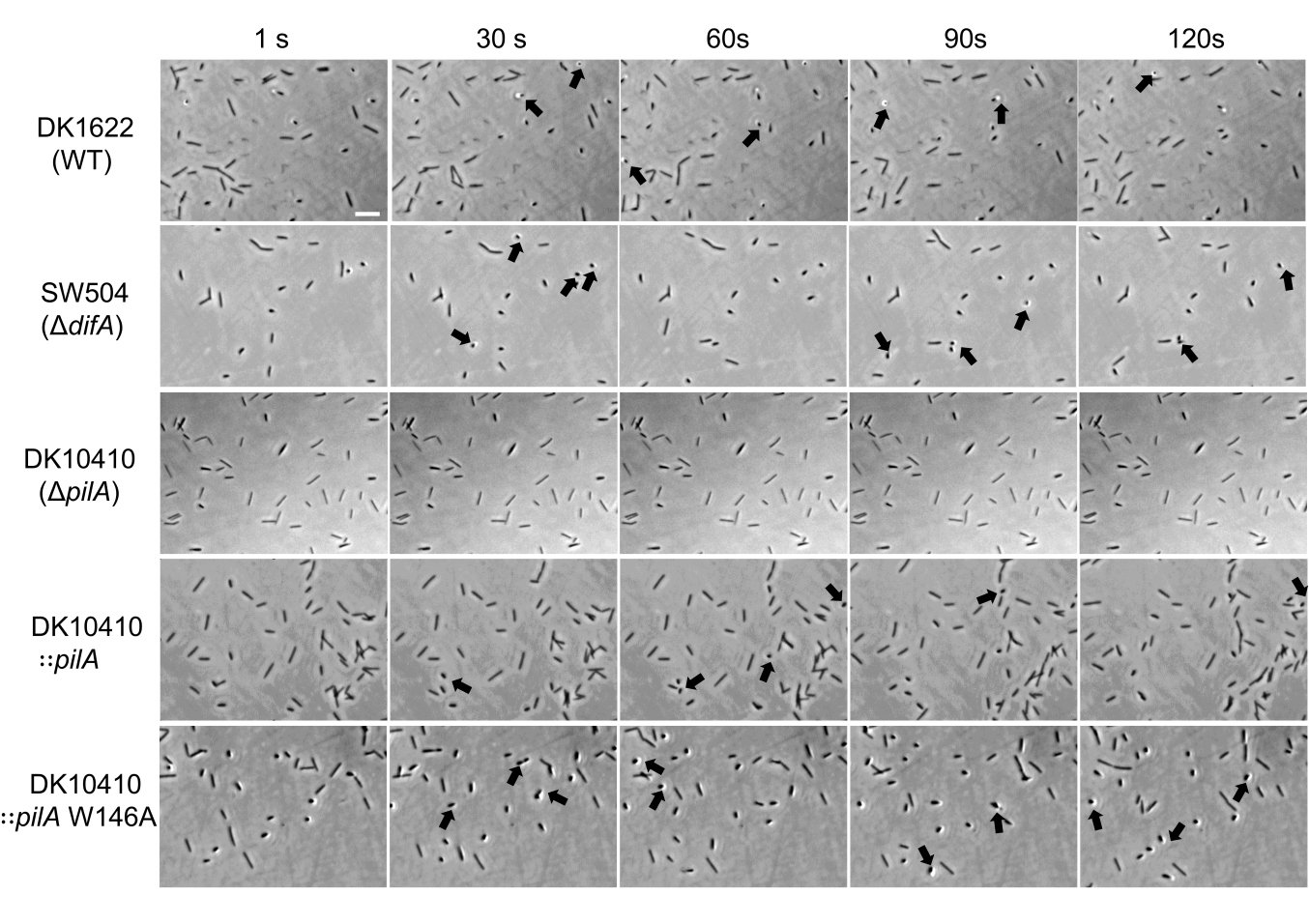
**

**Fig S13. PilA-W146A retains T4aP-mediated surface tethering behaviour when submerged in a 1% methylcellulose solution.** Under the 1% methylcellulose submerged conditions, the tethered cells appear as dots (denoted by black arrows). Sequential images are captured at 30-second intervals from left to right. With the assistance of T4aP, *M. xanthus* cells alternate between tethering and 'lying down' states. The scale bar represents 10 μm.

**
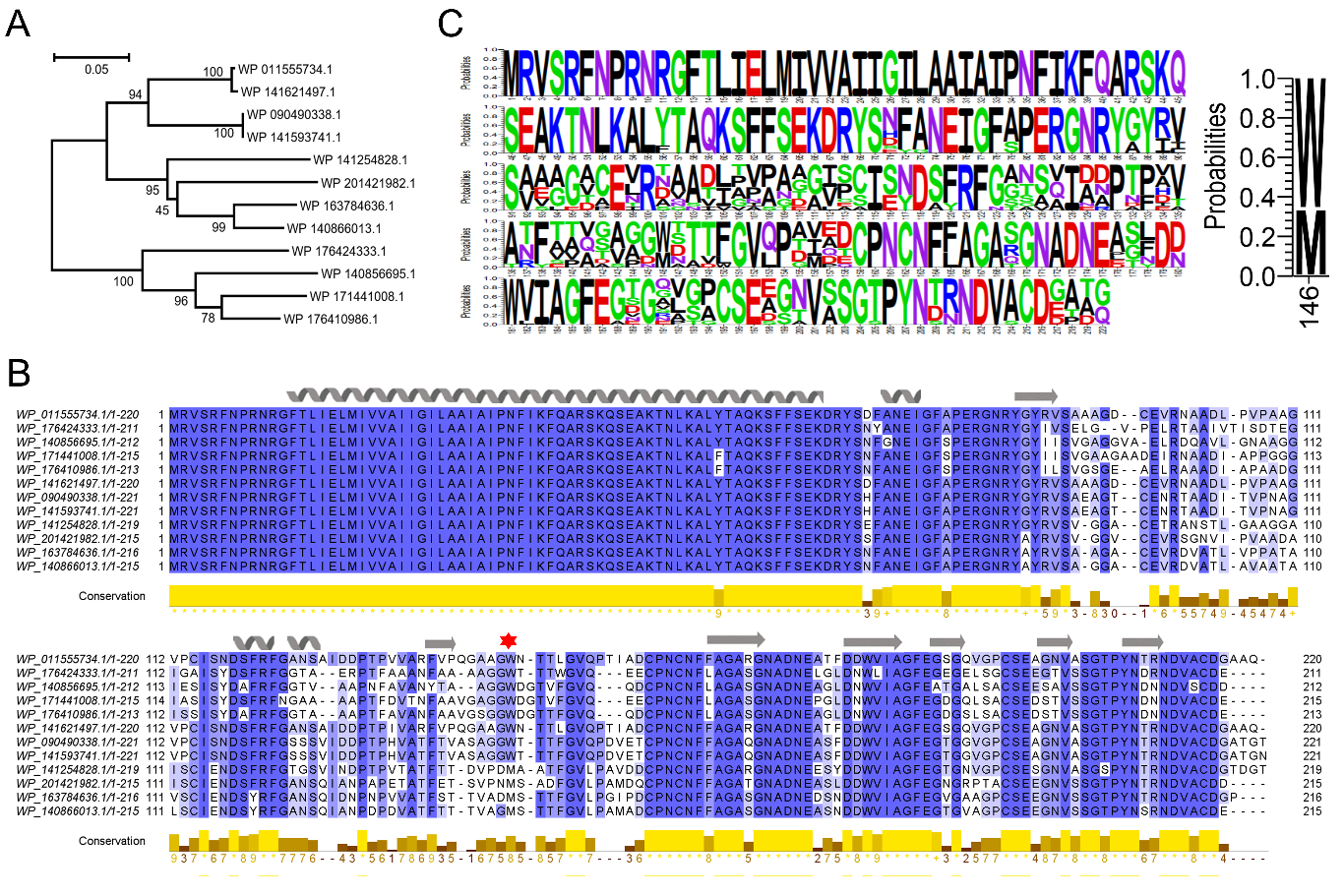
**

**Fig S14. Phylogenetic and conservation analysis of PilA residues.** (A) Phylogenetic tree of PilA proteins constructed using the neighbor-joining method based on homologous amino acid sequences from *Myxococcus* species. Branch numbers indicate bootstrap support values derived from 1000 replicates. (B) Multiple sequence alignment of PilA homologs. Alignment was performed using Clustal Omega and visualized in Jalview. Residues are shaded in blue according to conservation levels, with bright yellow highlighting positions of high conservation. Secondary structural elements are annotated above the alignment. (C) Sequence logo plot illustrating residue conservation in *Myxococcus* PilA proteins. The height of each letter corresponds to the relative frequency of the amino acid at that position, with taller letters indicating higher conservation.

**Table S1. Bacterial strains, plasmids, and primers used in this study.**

| **Strains, plasmids, and primers** | **Genotype, features, or sequence (5’-3’) description** |
| --- | --- |
| **Strains** |  |
| *Myxococcus xanthus* |  |
| DK1622 | Wild-type strain |
| DK10410 | DK1622 Δ*pilA* |
| DK10410::*pilA* | DK10410 site-specific integrated by plasmid pSWU-*pilA*; Kan^r^ |
| DK10410::*pilA* W146A | DK10410 site-specific integrated by plasmid pSWU-*pilA* W146A; Kan^r^ |
| DK10410::*pilA* W146Y | DK10410 site-specific integrated by plasmid pSWU-*pilA* W146Y; Kan^r^ |
| DK10410::*pilA* W146F | DK10410 site-specific integrated by plasmid pSWU-*pilA* W146F; Kan^r^ |
| DK10410::*pilA* W146G | DK10410 site-specific integrated by plasmid pSWU-*pilA* W146G; Kan^r^ |
| DK10410::*pilA* W146V | DK10410 site-specific integrated by plasmid pSWU-*pilA* W146V; Kan^r^ |
| DK10410::*pilA* W146L | DK10410 site-specific integrated by plasmid pSWU-*pilA* W146L; Kan^r^ |
| DK10410::*pilA* W146I | DK10410 site-specific integrated by plasmid pSWU-*pilA* W146I; Kan^r^ |
| DK10410::*pilA* W146S | DK10410 site-specific integrated by plasmid pSWU-*pilA* W146S; Kan^r^ |
| DK10410::*pilA* W146T | DK10410 site-specific integrated by plasmid pSWU-*pilA* W146T; Kan^r^ |
| DK10410::*pilA* W146C | DK10410 site-specific integrated by plasmid pSWU-*pilA* W146C; Kan^r^ |
| DK10410::*pilA* W146M | DK10410 site-specific integrated by plasmid pSWU-*pilA* W146M; Kan^r^ |
| DK10410::*pilA* W146D | DK10410 site-specific integrated by plasmid pSWU-*pilA* W146D; Kan^r^ |
| DK10410::*pilA* W146E | DK10410 site-specific integrated by plasmid pSWU-*pilA* W146E; Kan^r^ |
| DK10410::*pilA* W146N | DK10410 site-specific integrated by plasmid pSWU-*pilA* W146N; Kan^r^ |
| DK10410::*pilA* W146Q | DK10410 site-specific integrated by plasmid pSWU-*pilA* W146Q; Kan^r^ |
| DK10410::*pilA* W146K | DK10410 site-specific integrated by plasmid pSWU-*pilA* W146K; Kan^r^ |
| DK10410::*pilA* W146R | DK10410 site-specific integrated by plasmid pSWU-*pilA* W146R; Kan^r^ |
| DK10410::*pilA* W146H | DK10410 site-specific integrated by plasmid pSWU-*pilA* W146H; Kan^r^ |
| DK10410::*pilA* W146P | DK10410 site-specific integrated by plasmid pSWU-*pilA* W146P; Kan^r^ |
| DK10410::*pilA* Y69A | DK10410 site-specific integrated by plasmid pSWU-*pilA* Y69A; Kan^r^ |
| MHX2265 | DK1622 Δ*aglZ* |
| SW2022 | DK1622 Δ*aglZ*Δ*pilA* |
| SW2022::*pilA* | SW2022 site-specific integrated by plasmid pSWU-*pilA*; Kan^r^ |
| SW2022::*pilA* W146A | SW2022 site-specific integrated by plasmid pSWU-*pilA* W146A; Kan^r^ |
| SW504 | DK1622 Δ*difA* |
| SW2017 | DK1622 Δ*difA*Δ*pilA* |
| SW2017::*pilA* | SW2017 site-specific integrated by plasmid pSWU-*pilA*; Kan^r^ |
| SW2017::*pilA* W146A | SW2017 site-specific integrated by plasmid pSWU-*pilA* W146A; Kan^r^ |
| DK10409 | DK1622 Δ*pilT* |
| HW2201 | DK1622 Δ*pilT*Δ*pilA* |
| HW2201::*pilA* | HW2201 site-specific integrated by plasmid pSWU-*pilA*; Kan^r^ |
| HW2201::*pilA* W146A | HW2201 site-specific integrated by plasmid pSWU-*pilA* W146A; Kan^r^ |
| DK1622-mCherry | Fluorescence stain, DK1622 + pZJY4111-mCherry; Apra^r^ |
| DK1622-eGFP | Fluorescence stain, DK1622 + pZJY4111-eGFP; Apra^r^ |
| DK10410-eGFP | Fluorescence stain, DK10410 + pZJY4111-eGFP; Apra^r^ |
| SW504-eGFP | Fluorescence stain, SW504 + pZJY4111-eGFP; Apra^r^ |
| DK10410::*pilA*-eGFP | Fluorescence stain, DK10410::*pilA* + pZJY4111-eGFP; Apra^r^ |
| DK10410::*pilA* W146A-eGFP | Fluorescence stain, DK10410::*pilA* W146A + pZJY4111-eGFP; Apra^r^ |
| *Escherichia coli* |  |
| DH5α | F- endA1 glnV44 thi-1 recA1 relA1 gyrA96 deoR nupG 80dlacZΔM15Δ(lacZYA-arg F) U169, hsdR17(rK–mK+), λ– |
| BL21(DE3) | F^-^ompT hsdS_B_(r_B_^-^m_B_^-^) gal dcm (DE3) |
| XL1-Blue MR | Δ(mcrA)183Δ(mcrCB-hsdSMR-mrr)173 endA1 supE44 thi-1 recA1 gyrA96 relA1 lac |
| DH5α λpir | Φ80dlacZΔM15 ΔlacU169 recA1 endA1 hsdR17 supE44 thi-1 gyrA relA1 λpir |
| **Plasmids** |  |
| pBJ113 | Gene replacement vector with KG cassette; Kan^r^ |
| pBJ-*difA* | Upstream and downstream homologous arms of DK1622 *difA* inserted into the EcoRI and BamHI sites of pBJ113 |
| pBJ-*aglZ* | Upstream and downstream homologous arms of DK1622 *aglZ* inserted into the EcoRI and BamHI sites of pBJ113 |
| pBJ-*pilT* | Upstream and downstream homologous arms of DK1622 *pilT* inserted into the EcoRI and BamHI sites of pBJ113 |
| pSWU19 | Site-specific integration vector containing an Mx8 *attP* locus; Kan^r^ |
| pSWU-*pilA* | *pilA* promoter and PilA nucleotide sequences inserted into the XbaⅠ and EcoRⅠ sites of pSWU19 |
| pSWU-*pilA* W146A | *pilA* promoter and PilAW146A mutant nucleotide sequences inserted into the XbaⅠ and EcoRⅠ sites of pSWU19 |
| pSWU-*pilA* W146Y | *pilA* promoter and PilAW146Y mutant nucleotide sequences inserted into the XbaⅠ and EcoRⅠ sites of pSWU19 |
| pSWU-*pilA* W146F | *pilA* promoter and PilAW146F mutant nucleotide sequences inserted into the XbaⅠ and EcoRⅠ sites of pSWU19 |
| pSWU-*pilA* W146G | *pilA* promoter and PilAW146G mutant nucleotide sequences inserted into the XbaⅠ and EcoRⅠ sites of pSWU19 |
| pSWU-*pilA* W146V | *pilA* promoter and PilAW146V mutant nucleotide sequences inserted into the XbaⅠ and EcoRⅠ sites of pSWU19 |
| pSWU-*pilA* W146L | *pilA* promoter and PilAW146L mutant nucleotide sequences inserted into the XbaⅠ and EcoRⅠ sites of pSWU19 |
| pSWU-*pilA* W146I | *pilA* promoter and PilAW146I mutant nucleotide sequences inserted into the XbaⅠ and EcoRⅠ sites of pSWU19 |
| pSWU-*pilA* W146S | *pilA* promoter and PilAW146S mutant nucleotide sequences inserted into the XbaⅠ and EcoRⅠ sites of pSWU19 |
| pSWU-*pilA* W146T | *pilA* promoter and PilAW146T mutant nucleotide sequences inserted into the XbaⅠ and EcoRⅠ sites of pSWU19 |
| pSWU-*pilA* W146C | *pilA* promoter and PilAW146C mutant nucleotide sequences inserted into the XbaⅠ and EcoRⅠ sites of pSWU19 |
| pSWU-*pilA* W146M | *pilA* promoter and PilAW146M mutant nucleotide sequences inserted into the XbaⅠ and EcoRⅠ sites of pSWU19 |
| pSWU-*pilA* W146D | *pilA* promoter and PilAW146D mutant nucleotide sequences inserted into the XbaⅠ and EcoRⅠ sites of pSWU19 |
| pSWU-*pilA* W146E | *pilA* promoter and PilAW146E mutant nucleotide sequences inserted into the XbaⅠ and EcoRⅠ sites of pSWU19 |
| pSWU-*pilA* W146N | *pilA* promoter and PilAW146N mutant nucleotide sequences inserted into the XbaⅠ and EcoRⅠ sites of pSWU19 |
| pSWU-*pilA* W146Q | *pilA* promoter and PilAW146Q mutant nucleotide sequences inserted into the XbaⅠ and EcoRⅠ sites of pSWU19 |
| pSWU-*pilA* W146K | *pilA* promoter and PilAW146K mutant nucleotide sequences inserted into the XbaⅠ and EcoRⅠ sites of pSWU19 |
| pSWU-*pilA* W146R | *pilA* promoter and PilAW146R mutant nucleotide sequences inserted into the XbaⅠ and EcoRⅠ sites of pSWU19 |
| pSWU-*pilA* W146H | *pilA* promoter and PilAW146H mutant nucleotide sequences inserted into the XbaⅠ and EcoRⅠ sites of pSWU19 |
| pSWU-*pilA* W146P | *pilA* promoter and PilAW146P mutant nucleotide sequences inserted into the XbaⅠ and EcoRⅠ sites of pSWU19 |
| pSWU-*pilA* Y69A | *pilA* promoter and PilAY69A mutant nucleotide sequences inserted into the XbaⅠ and EcoRⅠ sites of pSWU19 |
| pET15b | His tag; the pBR322 replicon, T7 promoter; Amp^r^ |
| pMXE01 | Truncated PilA (PilACt) cloned into the EcoRI and BamHI sites of pET15b |
| pMXE011 | Truncated PilA (PilACt) mutant Y69A cloned into the EcoRI and BamHI sites of pET15b |
| pMXE012 | Truncated PilA (PilACt) mutant W146A cloned into the EcoRI and BamHI sites of pET15b |
| pMXE013 | Truncated PilA (PilACt) mutant W181A cloned into the EcoRI and BamHI sites of pET15b |
| pZJY4111 | The stable *E. coli*- *M. xanthus* shuttle plasmid containing ori and par loci of pMF1; Apra^r^ |
| pZJY4111-mCherry | *mCherry* nucleotide sequences inserted into the pZJY4111; Apra^r^ |
| pZJY4111-eGFP | *eGFP* nucleotide sequences inserted into the pZJY4111; Apra^r^ |
| **Primers** |  |
| Δ*pilT* U-F | AAAACGACGGCCAGTGAATTCTGGGGCGCTTCCGGGTCA |
| Δ*pilT* U-R | AAATGCCCGTGGCGGGTTCAGGAGCCCTTCCTTCCC |
| Δ*pilT* D-F | TGAACCCGCCACGGGCATTTCG |
| Δ*pilT* D-R | CAGGTCGACTCTAGAGGATCCAGCGGGCGGGCCGCCGCT |
| Δ *difA* U-F | AAAACGACGGCCAGTGAATTCGCCACAAGGCGCTGGCGG |
| Δ *difA* U-R | TCTGGCTCATGGGTTGCTTTCGGGGGAT |
| Δ *difA* D-F | AAAGCAACCCATGAGCCAGACGCCCTCG |
| Δ *difA* D-R | CAGGTCGACTCTAGAGGATCCCACCCGGAAGATGACGTGC |
| Δ*aglZ* U-F | AAAACGACGGCCAGTGAATTCCACTGACGCCCCAGCAGC |
| Δ*aglZ* U-R | GAAGCCCTTTGTCCCGGA |
| Δ*aglZ* D-F | TTTCCGGGACAAAGGGCTTCCGGCGCCGGGATTCCGGC |
| Δ*aglZ* D-R | CAGGTCGACTCTAGAGGATCCTCAGCACCGGGCGGGGCA |
| Δ*pilA* U-F | AAAACGACGGCCAGTGAATTCAGCGGGACGGGCAAGGAA |
| Δ*pilA* U-R | TTCCACTGAAGGAATGCGAGGGGGGTCCTCAGAGAAGGTTG |
| Δ*pilA* D-F | CTCGCATTCCTTCAGTGGAATG |
| Δ*pilA* D-R | CAGGTCGACTCTAGAGGATCCCGACTGTCATGGCATCTCCTC |
| 19-*pilA*-F | CTAGTCTAGATGCCTGCAGGTCGACTCTAGATTACTGGGCCGCGCCGTC |
| 19-*pilA*-R | CGCAGCACGGGTCTTCACGAATTCGTAATCATGGTCATAGAATTCCGG |
| PilAW146A-F | TCGTGTTggcCCCGGCAGCGCCCTGGGGCACG |
| PilAW146A-R | TGCCGGGgccAACACGACCCTCGGTGTGCAGC |
| PilAW146Y-F | TCGTGTTataCCCGGCAGCGCCCTGGGGCACG |
| PilAW146Y-R | TGCCGGGtatAACACGACCCTCGGTGTGCAGC |
| PilAW146F-F | TCGTGTTgaaCCCGGCAGCGCCCTGGGGCACG |
| PilAW146F-R | TGCCGGGttcAACACGACCCTCGGTGTGCAGC |
| PilAW146G-F | TCGTGTTtccCCCGGCAGCGCCCTGGGGCACG |
| PilAW146G-R | TGCCGGGggaAACACGACCCTCGGTGTGCAGC |
| PilAW146V-F | TCGTGTTaacCCCGGCAGCGCCCTGGGGCACG |
| PilAW146V-R | TGCCGGGgttAACACGACCCTCGGTGTGCAGC |
| PilAW146L-F | TCGTGTTaagCCCGGCAGCGCCCTGGGGCACG |
| PilAW146L-R | TGCCGGGcttAACACGACCCTCGGTGTGCAGC |
| PilAW146I-F | TCGTGTTaatCCCGGCAGCGCCCTGGGGCACG |
| PilAW146I-R | TGCCGGGattAACACGACCCTCGGTGTGCAGC |
| PilAW146S-F | TCGTGTTggaCCCGGCAGCGCCCTGGGGCACG |
| PilAW146S-R | TGCCGGGtccAACACGACCCTCGGTGTGCAGC |
| PilAW146T-F | TCGTGTTggtCCCGGCAGCGCCCTGGGGCACG |
| PilAW146T-R | TGCCGGGaccAACACGACCCTCGGTGTGCAGC |
| PilAW146C-F | TCGTGTTacaCCCGGCAGCGCCCTGGGGCACG |
| PilAW146C-R | TGCCGGGtgtAACACGACCCTCGGTGTGCAGC |
| PilAW146M-F | TCGTGTTcatCCCGGCAGCGCCCTGGGGCACG |
| PilAW146M-R | TGCCGGGatgAACACGACCCTCGGTGTGCAGC |
| PilAW146D-F | TCGTGTTatcCCCGGCAGCGCCCTGGGGCACG |
| PilAW146D-R | TGCCGGGgatAACACGACCCTCGGTGTGCAGC |
| PilAW146E-F | TCGTGTTttcCCCGGCAGCGCCCTGGGGCACG |
| PilAW146E-R | TGCCGGGgaaAACACGACCCTCGGTGTGCAGC |
| PilAW146N-F | TCGTGTTattCCCGGCAGCGCCCTGGGGCACG |
| PilAW146N-R | TGCCGGGaatAACACGACCCTCGGTGTGCAGC |
| PilAW146Q-F | TCGTGTTttgCCCGGCAGCGCCCTGGGGCACG |
| PilAW146Q-R | TGCCGGGcaaAACACGACCCTCGGTGTGCAGC |
| PilAW146K-F | TCGTGTTcttCCCGGCAGCGCCCTGGGGCACG |
| PilAW146K-R | TGCCGGGaagAACACGACCCTCGGTGTGCAGC |
| PilAW146R-F | TCGTGTTtctCCCGGCAGCGCCCTGGGGCACG |
| PilAW146R-R | TGCCGGGagaAACACGACCCTCGGTGTGCAGC |
| PilAW146H-F | TCGTGTTatgCCCGGCAGCGCCCTGGGGCACG |
| PilAW146H-R | TGCCGGGcatAACACGACCCTCGGTGTGCAGC |
| PilAW146P-F | TCGTGTTcggCCCGGCAGCGCCCTGGGGCACG |
| PilAW146P-R | TGCCGGGccgAACACGACCCTCGGTGTGCAGC |
| PilAY69A-F | AAGTCGGAggcACGGTCCTTCTCGGAGAAGAA |
| PilAY69A-R | GACCGTgccTCCGACTTCGCCAACGAAATCGG |
| PilAW181A-F | ATCACggcGTCATCGAAGGTCGCCTCGTTGTC |
| PilAW181A-R | TTCGATGACgccGTGATCGCCGGTTTCGAGGG |
| *mCherry*-F | AGAAAGAGGAGAAATACTAGATGGTGAGCAAGGGCGAGGAGGAT |
| *mCherry*-R | ATGCCTGGAAAAAATCACTTGTACAGCTCGTCCATGCCG |
| *eGFP*-F | AGAAAGAGGAGAAATACTAGATGGTGAGCAAGGGCGAGGAGCTGT |
| *eGFP*-R | ATGCCTGGAAAAAAGAATTCTCACTTGTACAGCTCATCCATGCCGA |

**Table S2 . AutoDock Vina results of the binding affinity and RMSD values of chitosan docked into PilA.**

| Model | Affinity (kcal/mol) | Distance from best mode (Å) | |
| --- | --- | --- | --- |
|  |  | RMSD l.b. | RMSD u.b. |
| Ⅰ | -6.2 | 0.000 | 0.000 |
| Ⅱ | -5.6 | 1.659 | 3.099 |
| Ⅲ | -5.5 | 3.021 | 13.374 |
| Ⅳ | -5.4 | 10.113 | 21.415 |
| Ⅴ | -5.4 | 1.591 | 2.484 |
| Ⅵ | -5.4 | 4.821 | 10.770 |
| Ⅶ | -5.3 | 1.930 | 2.843 |
| Ⅷ | -5.3 | 3.583 | 16.767 |
| Ⅸ | -5.2 | 4.668 | 17.345 |
| Ⅹ | -5.2 | 4.248 | 9.634 |

**Movie S1.** MD simulation of the PilA-glucosamine complex.

**Movie S2.** MD simulation of the PilA-glucose complex.

**Movie S3.** MD simulation of the PilA-N-Acetylglucosamine complex.

**Movie S4.** MD simulation of the PilA W146A-glucosamine complex.
